# Magnetoelectric effect generated through electron transfer from organic radical to metal ion

**DOI:** 10.1093/nsr/nwad059

**Published:** 2023-03-06

**Authors:** Xiaolin Liu, Qiang Liu, Haixia Zhao, Guilin Zhuang, Yanping Ren, Tao Liu, Lasheng Long, Lansun Zheng

**Affiliations:** Collaborative Innovation Center of Chemistry for Energy Materials, State Key Laboratory of Physical Chemistry of Solid Surfaces and Department of Chemistry, College of Chemistry and Chemical Engineering, Xiamen University, Xiamen 361005, China; State Key Laboratory of Fine Chemicals, Dalian University of Technology, Dalian 116024, China; Collaborative Innovation Center of Chemistry for Energy Materials, State Key Laboratory of Physical Chemistry of Solid Surfaces and Department of Chemistry, College of Chemistry and Chemical Engineering, Xiamen University, Xiamen 361005, China; College of Chemical Engineering, Zhejiang University of Technology, Hangzhou 310032, China; Collaborative Innovation Center of Chemistry for Energy Materials, State Key Laboratory of Physical Chemistry of Solid Surfaces and Department of Chemistry, College of Chemistry and Chemical Engineering, Xiamen University, Xiamen 361005, China; State Key Laboratory of Fine Chemicals, Dalian University of Technology, Dalian 116024, China; Collaborative Innovation Center of Chemistry for Energy Materials, State Key Laboratory of Physical Chemistry of Solid Surfaces and Department of Chemistry, College of Chemistry and Chemical Engineering, Xiamen University, Xiamen 361005, China; Collaborative Innovation Center of Chemistry for Energy Materials, State Key Laboratory of Physical Chemistry of Solid Surfaces and Department of Chemistry, College of Chemistry and Chemical Engineering, Xiamen University, Xiamen 361005, China

**Keywords:** molecular ferroelectric, electron transfer, radical, magnetoelectric effect

## Abstract

Magnetoelectric (ME) materials induced by electron transfer are extremely rare. Electron transfer in these materials invariably occurs between the metal ions. In contrast, ME properties induced by electron transfer from an organic radical to a metal ion have never been observed. Here, we report the ME coupling effect in a mononuclear molecule-based compound [(CH_3_)_3_NCH_2_CH_2_Br][Fe(Cl_2_An)_2_(H_2_O)_2_] (**1**) [Cl_2_An = chloranilate, (CH_3_)_3_NCH_2_CH_2_Br^+^ = (2-bromoethyl)trimethylammonium]. Investigation of the mechanism revealed that the ME coupling effect is realized through electron transfer from the Cl_2_An to the Fe ion. Measurement of the magnetodielectric (MD) coefficient of **1** indicated a positive MD of up to ∼12% at 10^3.^^0^ Hz and 370 K, which is very different from that of ME materials with conventional electron transfer for which the MD is generally negative. Thus, the current work not only presents a novel ME coupling mechanism, but also opens a new route to the synthesis of ME coupling materials.

## Teaser

The magnetoelectric coupling effect in molecular-based materials is realized for the first time by using the electron transfer of organic free radicals to metal ions.

## INTRODUCTION

Magnetoelectric (ME) materials, which undergo mutual transformation of the magnetic moment and electric polarization under applied magnetic and electric fields [1–3], are regarded as promising materials for a new generation of devices [4,5]. The first magnetodielectric (MD) coupling material, reported in 1960, was single-phase Cr_2_O_3_ [6]. The magnetic and electric orders are mutually exclusive in single-phase materials and such materials still remain extremely rare [7]. Most MEs are inorganic oxides such as LuFe_2_O_4_ [8,9], perovskite-type TbMnO_3_ and BiMnO_3_ [10,11], (Tb, Dy, Ho)Mn_2_O_5_ and Mn_2_FeSbO_6_ [12–14]. Thus, the exploration of new types of single-phase ME coupling materials is of key importance for their application.

In compounds that undergo valence tautomerism induced by electron transfer, the spin states and the coupling interactions change and the symmetry of the charge distribution also changes, resulting in a drastic change in both the magnetic and electric properties [15,16]. Although the electron-transfer-induced change in the spin transition and magnetic interaction has been extensively investigated [17,18], the induction of electric properties by electron transfer [19,20], especially ME coupling effects, has hardly been explored, despite a previous study that distinctly indicated that electron transfer between metal ions could result in ferroelectricity and a ME coupling effect [21–23]. On the other hand, although the ferroelectricity and ME coupling effect induced by electron transfer have been investigated, the ferroelectricity and ME coupling effects in these materials are always generated by electron transfer between metal ions. In contrast, direct electron transfer from an organic radical to a metal ion to generate the ME coupling effect has not been investigated yet. Here, we report the observation of ME effect in a mononuclear compound, [(CH_3_)_3_NCH_2_CH_2_Br][Fe(Cl_2_An)_2_(H_2_O)_2_] (**1**) [Cl_2_An = chloranilate, (CH_3_)_3_NCH_2_CH_2_Br^+^ = (2-bromoethyl)trimethylammonium].

## RESULTS AND DISCUSSION

### Crystal structures

The slow diffusion of chloranilic acid in acetone into an aqueous solution of FeCl_2_•4H_2_O and (2-bromoethyl)trimethylammonium bromide yielded black bulk crystals of **1** suitable for X-ray diffraction. The phase purity of **1** was confirmed by using powder X-ray diffraction (PXRD) analysis (Supplementary Fig. S1a). Thermogravimetric analysis (TGA) indicated that **1** is stable at ≤420 K in air atmosphere (Supplementary Fig. S1b).

**Figure 1.**
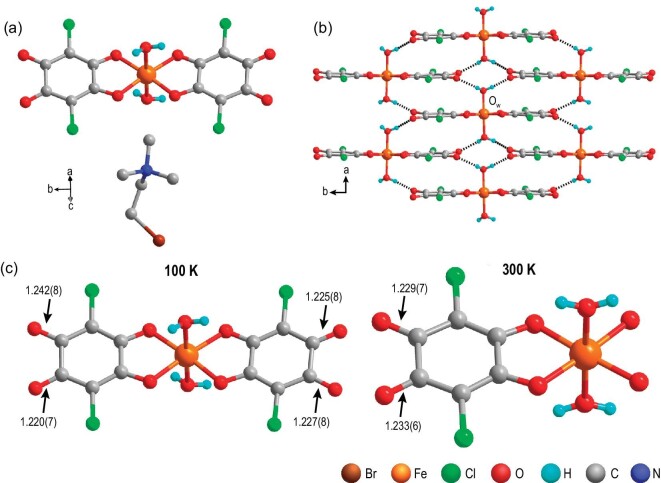
(a) Crystal structure of **1** at 100 K. H atoms of the (CH_3_)_3_NCH_2_CH_2_Br^+^ cation are omitted for clarity. (b) Layer structure of [Fe(Cl_2_An)_2_(H_2_O)_2_]^−^ (dashed lines denote hydrogen bonds). (c) Structures of [Fe(Cl_2_An)_2_(H_2_O)_2_]^−^ at 100 K (left) and 300 K (right); C–O bond distances (Å) are indicated and the (CH_3_)_3_NCH_2_CH_2_Br^+^ cation is omitted for clarity.

Single-crystal X-ray diffraction analysis of the compound at 100 K (hereinafter referred to as the low-temperature phase and abbreviated as LTP) reveals that **1** crystallized in the monoclinic polar *P*2_1_ space group with the following cell parameters: *a* = 7.0655(2) Å, *b* = 17.0164(5) Å, *c* = 9.5010(2) Å, *β* = 102.308(3)° and *Z* = 2 (Supplementary Table S1). The asymmetric unit of **1** consists of a mononuclear [Fe(Cl_2_An)_2_(H_2_O)_2_]^−^ anion and [(CH_3_)_3_NCH_2_CH_2_Br]^+^ cation (Fig. 1a). The Fe center is coordinated by four O atoms from two Cl_2_An ligands and two water molecules in a distorted octahedral coordination environment. Two adjacent [Fe(Cl_2_An)_2_(H_2_O)_2_]^−^ anions connected by hydrogen-bonding interactions through water molecules from one [Fe(Cl_2_An)_2_(H_2_O)_2_]^−^ anion (where the water molecules act as proton donors) and Cl_2_An as a proton acceptor generates a 2D layer structure, viewed along the *ab* plane (Fig. 1b and Supplementary Fig. S2). The adjacent layer structures are further connected by the [(CH_3_)_3_NCH_2_CH_2_Br]^+^ cation through Coulomb interaction to form the 3D supramolecular structure of **1** (Supplementary Fig. S2). The Fe−O (Cl_2_An) distances (Fig. 1c and Supplementary Table S2) are in the range of 1.978−1.993 Å, while the Fe−O (water) distances are in the range of 2.041−2.050 Å. The Fe−O distances in **1** are comparable to those reported in a high-spin Fe^III^ complex [24]. The distance of 1.220−1.242 Å for the uncoordinated C−O is in agreement with the normal distance for the double bond in the carbonyl group of chloranilic acid [25]. The room-temperature infrared (IR) spectrum (Supplementary Fig. S3) displays two characteristic peaks at 1538 and 1628 cm^−1^, assigned to C−O and C=O stretching vibrations, respectively [26]. These results suggest that Cl_2_An adopts an *o*-quinone-like structure [25]. The hydrogen-bonding distances for Ow–H•••O are 2.637(7), 2.657(7), 2.682(7) and 2.666(7) Å, respectively.

The crystal structure of **1** at room temperature (hereinafter termed the high-temperature phase and abbreviated as HTP) revealed that it crystallized in the monoclinic *P*2_1_/*m* space group with *a* = 7.19440(10) Å, *b* = 16.9900(2) Å, *c* = 9.58450(10) Å, *β* = 102.153(2)° and *Z* = 2 (Supplementary Table S1 and Supplementary Fig. S2d). The asymmetric unit contains half of a [Fe(Cl_2_An)_2_(H_2_O)_2_]^−^ anion and [(CH_3_)_3_NCH_2_CH_2_Br]^+^ cation. In comparison with **1** in the LTP, the [(CH_3_)_3_NCH_2_CH_2_Br]^+^ cation in the HTP is disordered, with the bromine and carbon on the methylene located in two different positions. The Fe−O (Cl_2_An) bond distances were in the range of 1.985(4)−1.986(4) Å, while the Fe−O (water) bond distance was in the range of 2.037(6)−2.049(6) Å (Supplementary Table S2). The bond distance for the uncoordinated C−O was 1.229−1.233 Å (Fig. 1c); the Ow–H•••O hydrogen-bonding distances were 2.687(5) and 2.665(5) Å, respectively. All the bond distances in the HTP are comparable to those in the LTP.

### Electron paramagnetic resonance (EPR) spectroscopy

**Figure 2.**
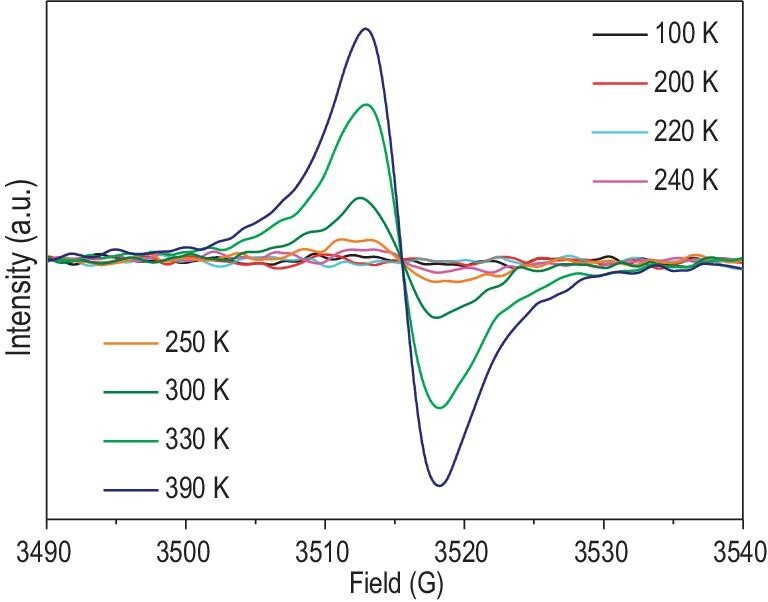
EPR spectra of **1** in the temperature range of 100−390 K.

Although all the bond distances in the HTP are comparable to those in the LTP, the EPR data for **1** acquired in the temperature range of 100−390 K displayed a significant temperature-dependent behavior. As illustrated in Fig. 2 and Supplementary Fig. S4, the EPR spectra of **1** display two signals centered at 3515 G (*g* = 2.0026) and 1640 G (*g* = 4.3), respectively. When the temperature was reduced to 220 K, the EPR signal centered at 3515 G gradually lost intensity and disappeared at <220 K. The signal centered at 3515 G with very narrow peak-to-peak line width (*ΔHpp*) (∼5 G) obtained from the EPR spectrum, along with the fact that the chloranilic acid ligand possesses multiple oxidation states (Supplementary Fig. S5) [27–29], indicates that the unpaired electron is located on the organic ligand [30–32]. This is consistent with the observation that the room-temperature solid-state UV–vis–NIR diffuse reflectance spectrum of **1** exhibits continuous intense absorption bands at higher *ν*_max_ values of between 23 000 and 41 000 cm^−1^ (Supplementary Fig. S6). This result reveals that some of the Cl_2_An ligands in **1** are present in the radical *o*-quinone form of Cl_2_An^−•^ and some of the Fe^III^ in **1** was reduced to Fe^II^ at 300 K. Based on the EPR signal centered at 1640 G, *ΔH*pp = 80 G were obtained (Supplementary Fig. S4), demonstrating that the Fe^III^ in **1** is in the high-spin (*hs*) state [33,34]. Therefore, at >220 K, some fraction of the Cl_2_An in **1** is in the *o*-quinone radical form of Cl_2_An^−•^, whereas at <220 K, the Cl_2_An in **1** is in the *o*-quinone form of Cl_2_An^2−^.

### Mössbauer spectra and X-ray photoelectron spectroscopy

To further confirm the existence of the Fe^III^–Cl_2_An^2−^ to Fe^II^–Cl_2_An^−•^ transition in **1**, the temperature-dependent Mössbauer spectra and X-ray photoelectron spectroscopy (XPS) of **1** were performed in the temperature range from 100 to 370 K, respectively. As shown in Supplementary Table S3 and Supplementary Fig. S7, fitting the Mössbauer spectra with a valence tautomerism (electron-transfer relaxation) model indicates that **1** at 370 K contains 87% of Fe^III^*_hs_* and 13% Fe^II^*_hs_*, and the content of the Fe^II^*_hs_* decreases with decreasing temperature [35]. At 100 K, the spectrum of **1** presents a symmetric doublet of Fe^III^*_hs_* and its symmetric doublet quadrupole splitting is 1.32 mm/s, indicating that only Fe^III^ exists in **1** at 100 K. Consistently, XPS (Supplementary Fig. S8a) at 100 K shows two peaks at 710.2 and 723.9 eV for the core-level spectrum of Fe (2*p*), which are assigned to Fe^III^ 2*p*_3/2_ and Fe^III^ 2*p*_1/2_, respectively. With increasing temperature, the two peaks move toward a lower binding energy direction and, at 370 K, two peaks at 709.28 and 722.92 eV for the core-level spectrum of Fe (2*p*) appeared, respectively, indicating the existence of Fe^II^ [36]. Based on the results of EPR, Mössbauer spectra and XPS, it is clear that a transition from Fe^III^–Cl_2_An^2–^ to Fe^II^–Cl_2_An^−•^ induced by electron transfer exists in **1**.

### Heat capacity

The heat capacity of **1** showed two hump peaks appear on the *C*_p_/*T–T* curve (Supplementary Fig. S8b). One is at ∼222 K and the other is at ∼235 K. Combining the single-crystal structures and EPR of **1** at different temperatures, the two peaks correspond to the phase transition of ordered–disordered ammonium and electron transfer, respectively.

### Magnetic and electric properties

**Figure 3.**
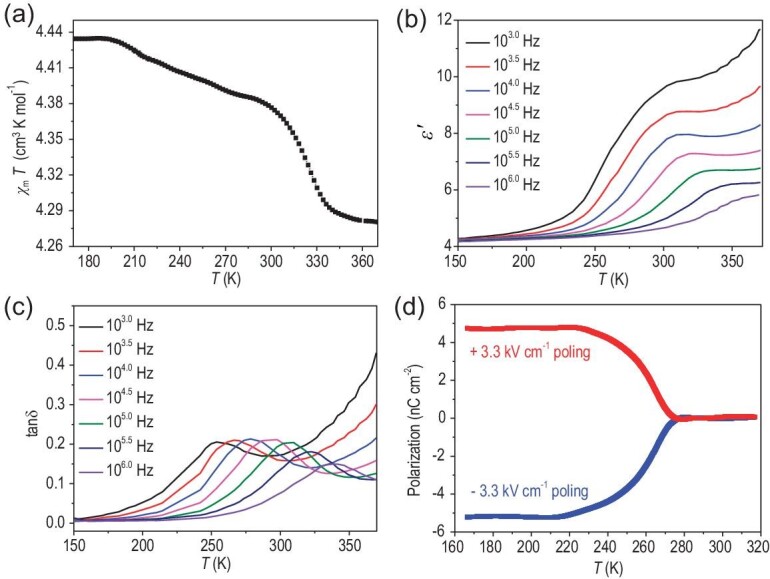
(a) Temperature-dependent magnetic susceptibility of **1** from 170 to 370 K. Temperature-dependence of (b) dielectric constant and (c) dielectric loss at different frequencies. (d) Temperature-dependent electric polarization of **1**.

Variable-temperature magnetic susceptibility (*χ*) measurements were performed on the powder samples of **1** under a *dc* field of 0.5 T (Supplementary Fig. S9a). Fitting the data in the temperature range of 100−200 K to the Curie–Weiss law gives *C* = 4.406 cm^3^ K mol^−1^ and *θ* = 1.194 K (Supplementary Fig. S9b). The small positive *θ* value indicates weak ferromagnetic coupling in **1**. As is shown in Fig. 3a and Supplementary Fig. S9, at 100 K, the experimental value of χ_M_T is 4.434 cm^3^ K mol^−1^, which is close to the theoretical value of 4.375 cm^3^ K mol^−1^ for a single Fe^III^*_hs_* (S = 5/2) ion. Upon increasing the temperature to ∼200 K, the experimental χ_M_T is ∼4.434 cm^3^ K mol^−1^. Owing to the generation of ∼4% Fe^II^ in this case, the theoretical χ_M_T value is 4.335 cm^3^ K mol^−1^ based on 96% Fe^III^*_hs_* (*S* = 5/2), 4% Fe^II^*_hs_* (*S* = 2) and 4% Cl_2_An^−•^ (*S* = 1/2), which is very consistent with the experimental one. At 370 K, the experimental χ_M_T is ∼4.280 cm^3^ K mol^−1^, in agreement with the theoretical value of 4.244 cm^3^ K mol^−1^ based on 87% Fe^III^*_hs_* (*S* = 5/2), 13% Fe^II^*_hs_* (*S* = 2) and 13% Cl_2_An^−•^ (*S* = 1/2) obtained from the temperature-dependent Mössbauer spectra of **1**.

Figure 3b and c shows the temperature- and frequency-dependent dielectric properties of **1** measured using a pressed pellet. The dielectric constant (ϵ* = *ϵ′* − *iϵ″*) is independent of the application frequency and remained almost constant (∼4) at <220 K. Increasing the temperature to >220 K induced a rapid increase in ϵ′ in a step-like manner. The shift of the dielectric peaks from 300 K (1 kHz) to 360 K (1 MHz) and the change in the peak position from 9.6 (1 kHz) to 5.7 (1 MHz) are typical of the dielectric relaxation process, as observed in [NH_2_(CH_3_)_2_][Fe^III^Fe^II^_(1–x)_Ni^II^_x_(HCOO)_6_] (x ≈ 0.63–0.69) and La_2_NiMnO_6_ [21,37]. The dielectric loss of **1** (Fig. 3c) also displayed similar behavior. Notably, the starting temperature point for the dielectric anomaly of **1** is very consistent with that for the production of the radical. Therefore, the dielectric anomaly is mainly attributed to the formation of the radical. To further confirm that it is the formation of the radical that plays a key contribution to the dielectric anomaly in **1**, two compou-nds, [(CH_3_)_3_NCH_2_CH_2_Cl][Fe(Cl_2_An)_2_(H_2_O)_2_] (**2**) and [(CH)_3_NCH_3_NCH_2_CH_3_][Fe(Cl_2_An)_2_(H_2_O)_2_] (**3**), with very similar structures to **1**, were prepared, respectively. Single-crystal structural analysis reveals that **2** invariably crystallizes in the *P*2_1_/*m* space group in the temperature range from 100 to 300 K and the cation of [(CH_3_)_3_NCH_2_CH_2_Cl]^+^ is disorder in the temperature range, while **3** always crystallizes in the *Pca*2_1_ space group and the cation of [(CH)_3_NCH_3_NCH_2_CH_3_]^+^ is order in the temperature range from 100 to 300 K. However, the temperature- and frequency-dependent dielectric properties indicate that **2** and **3** not only exhibit similar dielectric behavior as observed in **1**, but also display similar temperature-dependence EPR as observed in **1** (Supplementary Figs S10–S13). Although these results can not completely exclude the contribution of order–disorder of the cations to the dielectric anomaly in **1**, it is clear that the formation of the radical plays a key contribution to the dielectric anomaly.

Based on the Arrhenius equation (where *f* is the characteristic relaxation frequency, *f_0_* is the pre-exponential factor, *E_a_* is the activation energy for the phase transition, *T_p_* is the temperature of an *ϵ″* peak and *k_B_* is the Boltzmann constant), the activation energy was calculated as 0.643 eV (Supplementary Fig. S14), which is consistent with the energy for the charge transfer between iron and chloranilic acid [38]:


\begin{eqnarray*} f = {f}_0 \cdot {\rm{exp}}\left( { - \frac{{{E}_a}}{{{k}_B{T}_p}}} \right). \end{eqnarray*}

Because the crystal structures acquired at different temperatures reveal that the space group of **1** changed from *P*2_1_/_m_ to *P*2_1_ and the temperature-dependent second harmonic generation (SHG) shows that the SHG signal is active below the temperature of the structure phase transition of **1** (Supplementary Fig. S15), **1** may be a potential ferroelectric according to the Aizu rule, represented as 2/*mF*2 [39]. Thus, the ferroelectric polarization was investigated by measuring the temperature-dependent pyroelectric current. As shown in Fig. 3d, at <220 K, the polarization was ±5 nC cm^−2^ under positive and negative poling electric fields of *E* = ±3.3 kV cm^−1^, respectively, demonstrating the ferroelectricity in **1**. When the temperature was increased, the polarization decreased significantly at 220 K, which is the temperature for magnetic interaction of a fraction of the Fe^III^*_hs_* and Cl_2_An^2–^ and consequent conversion to Fe^II^*_hs_* and Cl_2_An^−•^. This result indicates that the ferroelectricity in **1** is generated by electron transfer from the Cl_2_An radical to the Fe ion. It is worth mentioning that the polarization does not disappear even at 220 K, indicating that **1** may be a relaxor ferroelectric [40,41].

Only considering the polarization induced by the electron transfer between the organic radical and the metal ion, the polarization was estimated on the basis of the point charge model (Supplementary Fig. S16). The net negative charge was imposed on the mobile electrons on the Cl_2_An^−•^/Fe^II^ sites, while the positive charge was located on the center of the amine ion. The polarization along the [010] direction (*P*_[010]_) induced by electron transfer was the reversible spontaneous polarization. The estimated polarization was ∼65.9 nC cm^−2^, which is significantly larger than the experimental value (5 nC cm^−2^) based on the powder pellets (Fig. 3d). For comparison, the polarization of **3** with ordered cation [(CH)_3_NCH_3_NCH_2_CH_3_]^+^ was also performed based on its powder pellets and the polarization in **3** was very close to that in **1** (Supplementary Fig. S17). Furthermore, isostructural nonmagnetic complex [(CH_3_)_3_NCH_2_CH_2_Br][Al(Cl_2_An)_2_(H_2_O)_2_] (**4**) was synthesized. The single-crystal structure at different temperatures shows that it undergoes the order–disorder phase transition of organic ammonium [(CH_3_)_3_NCH_2_CH_2_Br]^+^ at ∼260 K. Supplementary Fig. S18a shows the order phase (250 K) and disorder phase structures (300 K). Because no obvious polarization signal was detected in **4** (Supplementary Fig. S18b), the contribution of order and disorder organic ammonium to polarization and ME is thus excluded.

Supplementary Fig. S19 shows the spin-polarized constraint DFT calculation for the path of the polarization reversal. Crystallographically, the polarized state of **1** features the Fe^III^–Cl_2_An^2−^ state, while the unpolarized state features the Fe^II^–Cl_2_An^−•^ state. At 100 K, **1** maintains the polarized state. As the temperature increases, the electron gradually transfers from Cl_2_An^2−^ to Fe^III^ and thereby resulting in the Fe^II^–Cl_2_An^−•^ state; At 220 K, the electron in Cl_2_An^2−^ moves to the Fe^III^ ion and achieves electron-transferring equilibrium due to the effect of thermodynamical perturbation. It is revealed that the polarized state of Fe^III^–Cl_2_An^2−^ undergoes an uphill process to attain the unpolarized state of the Fe^II^–Cl_2_An^−•^ state with the energy barrier of 0.705 eV.

### ME effect

**Figure 4.**
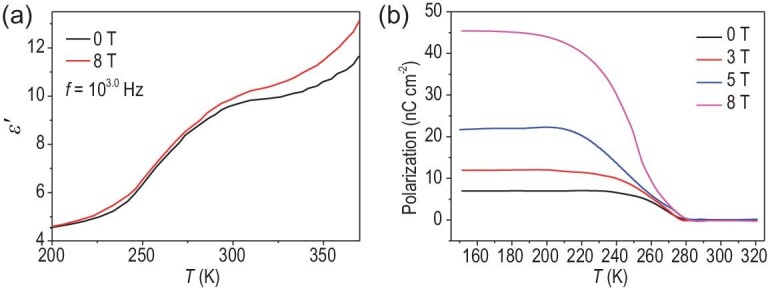
(a) Dielectric constant versus temperature at 10^3.^^0^ Hz, measured at zero field and under an external magnetic field of 8 T. (b) The electric polarization of **1** at different magnetic fields.

Owing to the shoulder in the electric polarization curve at the temperature of the onset of the magnetic transition, according to previous work [42], coupling of the magnetization with the electric polarization would be expected. Thus, the magnetic field dependence of *ϵ′* was investigated at different frequencies of **1** (Fig. 4a and Supplementary Fig. S20). As shown in Fig. 4a, in comparison with *ϵ′* at the zero field, at <220 K, *ϵ′* remained almost unchanged under an external magnetic field of 8 T. At >220 K, however, *ϵ′* became significantly higher under the external magnetic field. At 370 K, the MD coefficient {MD = [*ϵ′*(H) −*ϵ′*(0)]/*ϵ′*[0] = Δ*ϵ′/ϵ′*[0]} reached a maximum value of ∼12% at 10^3.^^0^ Hz. Significantly, the temperature-dependent pyroelectric current at different magnetic fields reveals that the polarization was significantly increased with increasing magnetic field (Fig. 4b); a conspicuous ME effect is clearly observed in **1**. It was mentioned that the ME of **3** exhibits similar behavior as observed in compound **1** (Supplementary Fig. S21). This result, together with the fact that no obvious polarization signal was detected in **4**, further demonstrates that the ME effect in **1** is induced by the electron transfer between the organic radical and the metal ion.

In order to exclude the contribution of the magnetoresistance originating from the grains and that of spin polarization to the MD in **1**, the dielectric loss of **1** under an external magnetic field of 8 T was evaluated using a pressed pellet (Supplementary Fig. S22). The increase in dielectric loss with increasing magnetic field, as well as the very low value of dielectric loss, confirmed the insulating nature of **1** and clearly excludes the contribution of the Maxwell–Wagner effect to the positive MD in **1** [43]. This demonstrates that the positive MD in **1** is intrinsic, because the magnetoresistance originating from the grains and spin polarization often generates a negative MD. In addition, the difference in the dielectric constant at 0 and 8 T is consistent with the thermally induced change due to charge transfer, which corroborates that the MD in **1** is related to the charge transfer to some extent.

Notably, five electron ferroelectrics have so far been reported to have ME effects; these are LuFe_2_O_4_, [(*n*-C_3_H_7_)_4_ N][Fe^III^Fe^II^(dto)_3_] (dto = C_2_O_2_S_2_), [Fe_3_O(CH_3_COO)_6_(py)_3_](py), [NH_2_(CH_3_)_2_][Fe^III^Fe^II^_(1–x)_Ni^II^_x_(HCOO)_6_] (x ≈ 0.63–0.69) and [Fe(2,2′-bipyridine)(CN)_4_]_2_Co-(4,4′-bipyridine)•4H_2_O [9,21–23,44]. For these species, the ferroelectricity is invariably produced through electron transfer between adjacent metal ions, while the ferroelectricity in **1** is generated by direct electron transfer from the organic radical ligand to the metal ion, which has never been observed before. On the other hand, although three electron ferroelectrics have so far been reported to exhibit MD effects, their MD is invariably negative.

Thus, **1** not only represents the first example that the ME coupling effect induced by electron transfer results in a positive MD, but also opens a new route to the synthesis of ME coupling materials.

### Theoretical calculations

To further confirm that the ME mechanism is related to electron transfer from the organic radical to the metal ion, spin-polarized theoretical calculations were performed (Supplementary data). First, multiconfigurational CASSCF/NEVPT2 calculation results reveal that, at 100 K, **1** with *C*_2h_ symmetry features a high-spin sextet state with the electronic configuration of (a_u_)^2^(b_u_)^2^(b_g_)^2^(a_g_)^1^(b_g_)^1^(a_u_)^1^(a_g_)^1^(a_u_)^1^, while, via the electron transfer at 230 K from double-occupied molecular orbitals (DOMOs) of a_u_ or b_u_ to the single-occupied molecular orbitals (SOMOs) of a_g_, **1** attains the quartet electronic configuration with the antiferromagnetic interaction of –102.64 cm^−1^ between the Cl_2_An^−•^ and Fe^II^ ion. Such degenerate transitions may lead to spin–orbit coupling between the Fe ion and the ligand [45]. Spin-density population reveals that the Cl_2_An^−•^ evenly distributes on four coordinated oxygen atoms (Supplementary Fig. S23). Furthermore, non-collinear DFT calculation results show that Cl_2_An^−•^ and Fe^II^ ion feature a spin-canting property (Supplementary Fig. S24 and Supplementary Table S4) with Dzyaloshinskii–Moriya (DM) interaction rather than spin antiparallel distribution [46]. Two quarter-spins of para-position oxygen atoms feature the same orientation, while those of ortho-position oxygen atoms almost keep the reverse orientation. Electric-field dependent DFT calculation results indicated an anisotropic effect on electronic properties (Supplementary Figs S23 and S25–S27), compatible with previously reported results [47]. The electric field along the [010] direction apparently promotes the electron transfer from the ligand to the Fe ion (Supplementary Figs S23 and S25b), resulting in the enhanced electronic polarization effect along the [010] direction (Supplementary Fig. S26). Compared with [010], [001] and [100] directions deviate from the polarization axis, and never possess the electronic polarization effect.

**Figure 5.**
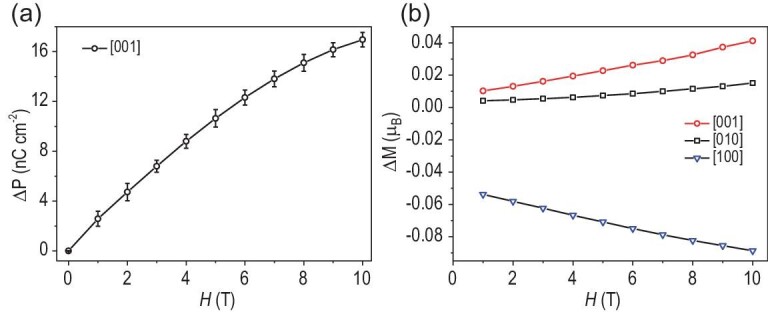
Applied magnetic field-induced electronic polarization. (a) Change of **1** with Fe^II^ state along [001]) and (b) change of orbital magnetic moment along three directions [001], [010] and [100].

Furthermore, the polarization response under the applied magnetic field was theoretically studied by using the self-consistent response to the Zeeman field for non-collinear spins. Computational results show that the electronic polarization response along the [001] lattice direction is linearly correlated with the magnetic field (Fig. 5a), where the electronic polarization increased ∼15.10 nC cm^−2^ at 8 T, consistently with the trend for Cr_2_O_3_ reported by Delaney [48], while along the [100] and [010] directions, the response degree of electronic polarization is almost ignored (Supplementary Fig. S28). Generally, such a difference can be attributed to the change in the orbital moment induced by spin–orbit coupling under the applied magnetic field [49]. A magnetic field along the [001] direction apparently promoted electronic transfer from anti-bonding DOMOs (a_u_ or b_u_) of the ligand to anti-bonding coordination orbital (a_g_) of **1**, resulting in the improving ratio of orbital-moment unquenching Fe^II^ ion (Fig. 5b), and thereby enhancing the whole orbital moment with the change of 0.033 μ_B_ at 8 T (0.246 μ_B_ at 0 T). Moreover, the resultant asymmetric a_u_ or b_u_ SOMO induces polarization according to the DM interaction property [46], where the corresponding obtained electronic polarization lies in the symmetry-arrowed polarization direction of [010] (Supplementary Figs S29 and S30), and thus produces an obvious ME effect. As the lattice direction of [100] is perpendicular to the phenyl of the ligand, the magnetic field does not enhance the orbital moment and, on the contrary, slightly reduces the orbital moment because of the coordination environment of the aqua ligand. Although the magnetic field along the [010] direction slightly enhanced the orbital moment (Fig. 5b), the resultant electronic polarization direction deviated from the symmetry-arrowed [010] polarization direction under *P*2_1_ symmetry and gave rise to the weak ME effect. These results, together with the intrinsic spontaneous polarization mechanism of **1**, indicate that the ME coupling in **1** is achieved by spin-mediated promoting electron transfer under the magnetic field, because the increasing short-range spin–spin interaction between the Fe ion and Cl_2_An^−•^ under a strong magnetic field will promote the electron transfer, and further lead to the increased polarization [50]. Therefore, it is concluded that the ME effect is induced by the non-collinear spin configurations between the Fe ion and the ligand under a magnetic field, and meanwhile anisotropic polarization is mainly associated with the local anisotropy of the coordination field and the restriction of crystallographic symmetry.

## CONCLUSIONS

In summary, the ME coupling effect induced by electron-transfer-induced valence tautomerism was observed in **1**. Investigation of the crystal structure of **1** at different temperatures reveals that **1** crystallized in the polar *P*2_1_ space group in the LTP and *P*2_1_/*m* space group in the HTP. Study of the temperature-dependent EPR and magnetism of **1** indicates that the formation of the HTP is due to transformation of a fraction of the Fe^III^ and Cl_2_An^2−^ in the LTP to Fe^II^ and Cl_2_An^−•^, respectively. Compound **1** exhibits a positive MD of >12% and its polarization is significantly increased under an external magnetic field. Spin-polarized computational calculations reveal that the ME coupling effect in **1** is mainly derived from spin–orbit coupling between Fe^II^ and Cl_2_An^−•^ under the magnetic field. A significant increase in the electronic polarization under the magnetic field is mainly due to the spin-mediated promoting electron transfer under the magnetic field. Considering the fact that the ferroelectricity generated via direct electron transfer from an organic radical to a metal ion has never been observed before, and a positive MD effect and ME have not yet been found in electron ferroelectric materials, the present work not only represents a novel ME coupling mechanism, but also opens a new route to the synthesis of ME coupling materials.

## Supplementary Material

nwad059_Supplemental_Files
