# Supplementary material for: Magnetoelectric effect generated through electron transfer from organic radical to metal ion
Source: Natl Sci Rev. 2023 Mar 6;10(4):nwad059. doi: 10.1093/nsr/nwad059 (PMC10187783; doi:10.1093/nsr/nwad059)
Supplement: nwad059_Supplemental_Files [file nwad059_supplemental_files.zip › Supplementary data.docx]

**Supplementary Data**

**Magnetoelectric Effect Generated through Electron transfer from Organic Radical to Metal Ion**

Xiaolin Liu,^1^ Qiang Liu,^3^ Haixia Zhao,^1^* Guilin Zhuang,^2^* Yanping Ren,^1^ Tao Liu,^3^ Lasheng Long^1^* and Lansun Zheng^1^

^1^Collaborative Innovation Center of Chemistry for Energy Materials, State Key Laboratory of Physical Chemistry of Solid Surfaces and Department of Chemistry College of Chemistry and Chemical Engineering, Xiamen University, Xiamen 361005, China

^2^College of Chemical Engineering, Zhejiang University of Technology, Hangzhou, 310032, China

^3^State Key Laboratory of Fine Chemicals, Dalian University of Technology, Dalian 116024, China

**Experimental**

All chemicals used in the synthesis were of reagent grade and were used without further purification.

Synthesis of **1**

An aqueous solution (6 mL) of FeCl_2_•4H_2_O (32 mg, 0.16 mmol) and (2-bromoethyl) trimethylammonium bromide (BCB) (40 mg, 0.16 mmol) was placed in a tube as the bottom layer, after which a mixture of acetone (2 mL) and water (2 mL) as a buffer layer was carefully placed on the bottom layer; finally an acetone (6 mL) solution of chloranilic acid (H_2_Cl_2_An) (25 mg, 0.12 mmol) as the top layer was placed on the top. After allowing the system to stand for 3 weeks, black block crystals were obtained at the bottom of the tube in 80% yield (based on FeCl_2_•4H_2_O). Elemental analysis (found (%): C 29.81, H 2.47, N 1.82; calcd. (%) for **1**: C 30.3, H 2.53, N 2.08).

Synthesis of **2**

The method of synthesis of **2** is similar to **1**, it's just use (2-Chloroethyl)trimethylammonium chloride (25 mg, 0.16 mmol) substitute for (2-bromoethyl) trimethylammonium bromide (BCB) (40 mg, 0.16 mmol). After allowing the system to stand for 3 weeks, black block crystals were obtained at the bottom of the tube in 75% yield (based on FeCl_2_•4H_2_O). Elemental analysis (found (%): C 32.26, H 2.69, N 2.27; calcd. (%) for **2**: C 32.49, H 2.73, N 2.23).

Synthesis of **3**

The method of synthesis of **3** is similar to **1**, it's just use 1-ethyl-3-methylimidazolium chloride (23 mg, 0.16 mmol) substitute for (2-bromoethyl) trimethylammonium bromide (BCB) (40 mg, 0.16 mmol). After allowing the system to stand for **3** weeks, black block crystals were obtained at the bottom of the tube in 61% yield (based on FeCl_2_•4H_2_O). Elemental analysis (found (%): C 34.90, H 2.51, N 4.59; calcd. (%) for **3**: C 35.04, H 2.45, N 4.54).

Synthesis of **4**

The method of synthesis of **4** is similar to **1**, it's just use AlCl_3_ (21 mg, 0.16 mmol) substitute for FeCl_2_•4H_2_O (32 mg, 0.16 mmol). After allowing the system to stand for **3** weeks, reddish black block crystals were obtained at the bottom of the tube in 73% yield (based on AlCl_3_). Elemental analysis (found (%): C 30.91, H 2.57, N 2.08; calcd. (%) for **4**: C 31.71, H 2.66, N 2.18).

**Physical measurement methods**

**Crystal structure determination**

X-ray single-crystal diffraction data were collected at different temperatures by using an Agilent Supernova CCD diffractometer in a *ω*-scan mode, with Δ*ω* = 1.0°. The single-crystal diffraction data were collected by using a Mo-K*α* radiation (*λ* = 0.71073 Å) source and Cu-K*α* radiation (*λ* = 1.5418 Å). Data collection and processing were accomplished with the CrysAlis PRO program. Absorption corrections were applied by using the multi-scan program. The structures were solved and refined using full matrix least-squares based on *F*^2^ with the SHELXS-2013/SHELXL-2013 programs within OLEX 2. Because the [(CH_3_)_3_NCH_2_CH_2_Br]^+^ cation is disordered, the hydrogen atoms on this cation were not added. The crystal data, as well as the details of data collection and refinement for the complexes, are summarized in Table S3. The supplementary crystallographic data for this paper are accessible at CCDC 1982773 (**1**-100 K), 1982774 (**1**-300 K), 2030861 (**1**-200 K), 2030866 (**1**-220 K), 2030871 (**1**-240 K), 2030875 (**1**-260 K), 2030878 (**1**-270 K), 2030882 (**1**-280 K), 2030812 (**2**-100 K), 2030842 (**2**-200 K), 2030852 (**2**-300 K), 2078116 (**3**-100 K), 2078117 (**3**-300 K), 2170347 (**4**-250 K), 2170349 (**4**-300 K). These data can be obtained free of charge from the Cambridge Crystallographic Data Centre.

**Measurement of electrical properties**

The temperature-dependent dielectric constants (*ε** = *ε*′ − i*ε*″) were measured by two-probe *a.c.* impedance analysis (Wayne Kerr 6500B Precise Impedance Analyzer). The electric contacts were prepared by using silver paste (DAD-87) to attach 50 μm gold wires to the pressed pellets of the pure samples. The pellets were placed into a Janis cryogenic refrigeration system equipped with an American Cryomagnetics 9 T Superconducting Magnet.

The pyroelectric current was measured with powder samples of **1**, **3** and **4** on a Keithley 6517B Electrometer/High Resistance Meter under zero-electric field after a poling procedure.

**Spectral acquisition**

Electron paramagnetic resonance (EPR) spectra were acquired with an X-band Bruker A 300 spectrometer operating at approximately 9.4 GHz.

X-ray photoelectron spectroscopy (XPS) data were acquired in an UHV chamber equipped with an Omicron XPS (base pressure 2 × 10^−10^ mBar) instrument with a monochromatic aluminum anode X-ray source that supplied K*α* radiation (1486.6 eV).

Mössbauer spectra were acquired by using a proportional counter and a Topologic 500A spectrometer with ^57^Co (Rh) as a γ-ray radioactive source.

Infrared spectra (IR) were recorded on a Nicolet AVATAR FT-IR 360 spectrophotometer with pressed KBr pellets.

Ultraviolet–visible–near infrared spectroscopy measurements were performed with a UV-Vis-NIR spectrophotometer (Cary 5000) at room temperature.

Variable-temperature Second harmonic generation (SHG) experiments of **1** were performed on powder samples using the femto-second laser (pulsed Nd:YAG at a wavelength of 1200 nm, 120 fs pulse duration, 1 kHz repetition rate).

**Measurement of other properties**

The magnetic susceptibility of the samples was measured with a Quantum Design MPMS superconducting quantum interference device (SQUID). A powder sample of **1** was analyzed under a *dc* field of 5000 Oe.

Heat capacity measurements were performed using a Quantum Design PPMS in the temperature range 100–300 K.

Powder X-ray diffraction (PXRD) patterns were recorded on a Rigaku Ultima IV diffractiometer with Cu-K*α* radiation (*λ* = 1.5418 Å) with a graphite monochromator.

C, H, and N microanalyses were carried out with a CE instruments EA 1110 elemental analyzer.

The thermogravimetric analysis (TGA) curves were obtained using a SDTQ 600 Thermal Analyzer.

Table S1. Crystallographic data for 1 at 100 K and 300 K.

| Compound | 1 | |
| --- | --- | --- |
| Temp (K) | 100.01(10) | 300.01(10) |
| formula weight | 672.87 | 672.87 |
| crystal system | monoclinic | monoclinic |
| space group | *P*2_1_ | *P*2_1_*/m* |
| *a*(Å) | 7.0655(2) | 7.19440(10) |
| *b*(Å) | 17.0164(5) | 16.9900(2) |
| *c*(Å) | 9.5010(2) | 9.58450(10) |
| *α* (deg) | 90 | 90 |
| *β* (deg) | 102.308(3) | 102.153(2) |
| *γ* (deg) | 90 | 90 |
| volume (Å^3^ ) | 1116.04(5) | 1145.29(3) |
| Z | 2 | 2 |
| Scan mode | *ω*-scan | *ω*-scan |
| *μ* (mm^−1^) | 3.002 | 12.139 |
| *F*(000) | 670.0 | 670.0 |
| crystal size (mm^3^) | 0.1 × 0.3× 0.1 | 0.1 × 0.5 × 0.05 |
| radiation | Mo K*α* | Cu K*α* |
| wavelength (Å) | 0.71073 | 1.54178 |
| 2*θ* range for data  collection (deg) | 6.986-60.808 | 9.44-130.352 |
| index ranges | −9 ≤ *h* ≤ 9,  −24 ≤ *k* ≤ 22,  −13 ≤ *l* ≤ 12 | −8 ≤ *h* ≤ 8,  −17 ≤ *k* ≤ 19,  −11 ≤ *l* ≤ 11 |
| density (calcd) (g cm^-3^) | 2.002 | 1.951 |
| reflns collected | 11360 | 6275 |
| *R*_int_ | 0.0398 | 0.0546 |
| Refinement method | full-matrix least-squares on  *F*^2^ | |
| data/restraints/parameters | 5780/1/312 | 2013/01/179 |
| Goodness-of-fit on *F*^2^ | 1.037 | 1.087 |
| *R*_1_, *ωR*_2_ [ I > 2*σ*(*I*)] | 0.0416, 0.0922 | 0.0665, 0.1793 |
| *R*_1_, *ωR*_2_ [ all data ] | 0.0473, 0.0968 | 0.0707, 0.1839 |
| *ρ*_max_, *ρ*_min_ (e Å^−3^ ) | 1.31, -0.89 | 0.74, -0.72 |
| ^a^R_1_ = ∑\|\|*F_o_*\| − \|*F_c_*\|\|/∑\|*F_o_*\|, *ω*R_2_ = {∑[I(\|*F_o_*\|^2^ − \|*F_c_*\|^2^)]/∑[*ω*\|*F_o_*\|^4^]}^1/2^ and *ω* = 1/[σ^2^(*F_o_*^2^) + (0.1361P)^2^] for HTP and *ω* = 1/[*σ*^2^(*F_o_*^2^) + (0.0611P)^2^ + 1.3669P] for LTP, where P = (*F_o_*^2^ + 2*F_c_*^2^)/3. | | |

**Table S2**. Selected Bond Lengths (Å) for **1**.

|  | 100 K | 300 K |
| --- | --- | --- |
| Fe1 – O1 (Å) | 1.978(4) | 1.986(4) |
| Fe1 – O2 (Å) | 1.983(4) | 1.985(4) |
| Fe1 – O3 (Å) | 1.993(4) | 1.986(4) |
| Fe1 – O4 (Å) | 1.993(4) | 1.985(4) |
| Fe1 – O5 (Å) | 2.041(3) | 2.049(6) |
| Fe1 – O6 (Å) | 2.050(4) | 2.037(6) |
| C1 – O2 (Å) | 1.296(8) | 1.287(6) |
| C2 – O1 (Å) | 1.288(8) | 1.277(7) |
| C4 – O7 (Å) | 1.242(8) | 1.229(6) |
| C5 – O8 (Å) | 1.220(7) | 1.233(6) |
| C7 – O4 (Å) | 1.271(7) | 1.287(6) |
| C8 – O3 (Å) | 1.281(7) | 1.277(7) |
| C10 – O9 (Å) | 1.227(8) | 1.229(6) |
| C11 – O10 (Å) | 1.225(8) | 1.233(6) |
| C1 – C2 (Å) | 1.504(9) | 1.520(8) |
| C2 – C3 (Å) | 1.386(9) | 1.366(7) |
| C3 – C4 (Å) | 1.416(9) | 1.425(7) |
| C4 – C5 (Å) | 1.563(9) | 1.554(9) |
| C5 – C6 (Å) | 1.422(9) | 1.428(7) |
| C6 – C1 (Å) | 1.371(9) | 1.372(7) |
| C7 – C8 (Å) | 1.532(8) | 1.520(8) |
| C8 – C9 (Å) | 1.367(8) | 1.366(7) |
| C9 – C10 (Å) | 1.227(8) | 1.425(7) |
| C10 – C11 (Å) | 1.556(9) | 1.554(9) |
| C11 – C12 (Å) | 1.419(9) | 1.428(7) |
| C12 – C7 (Å) | 1.371(9) | 1.372(7) |

**
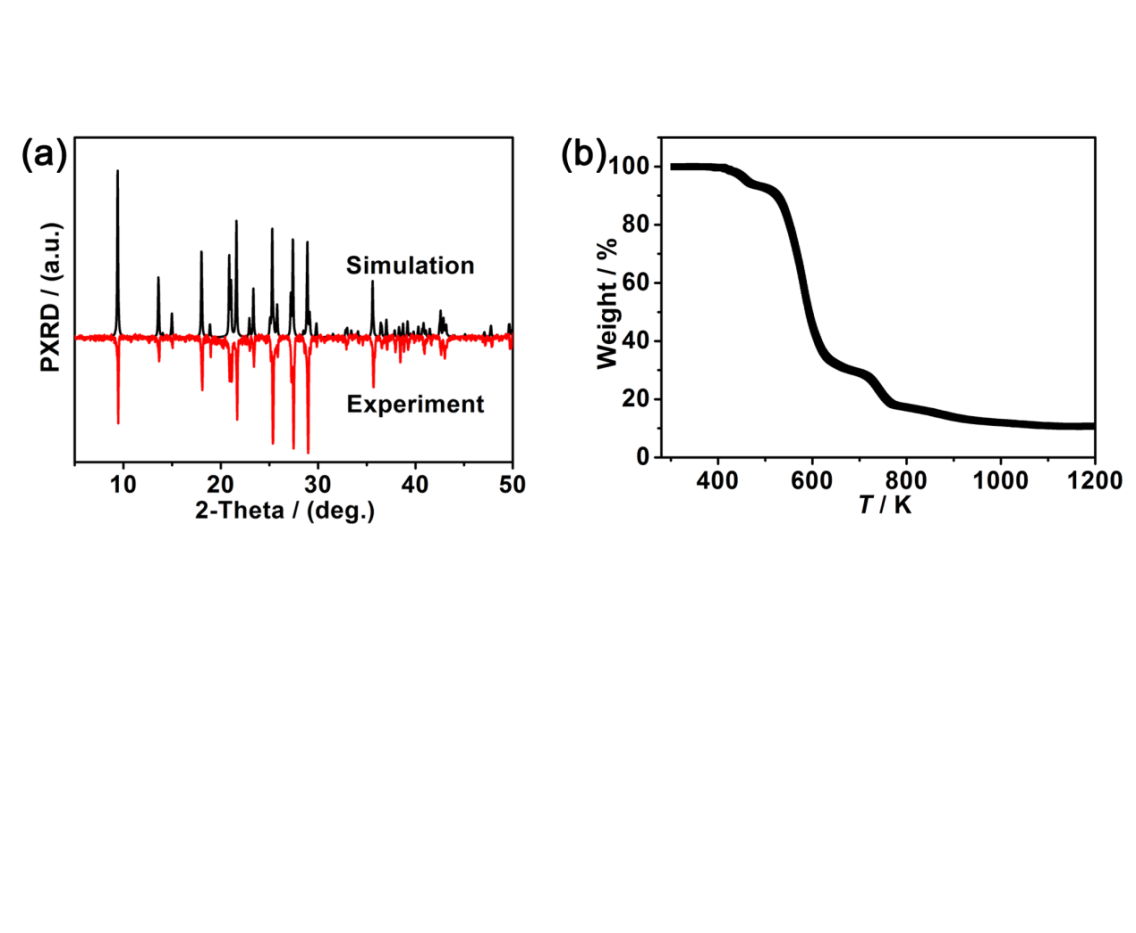
**

Figure S1. (a) PXRD patterns at room temperature and (b) TG curve of **1**.


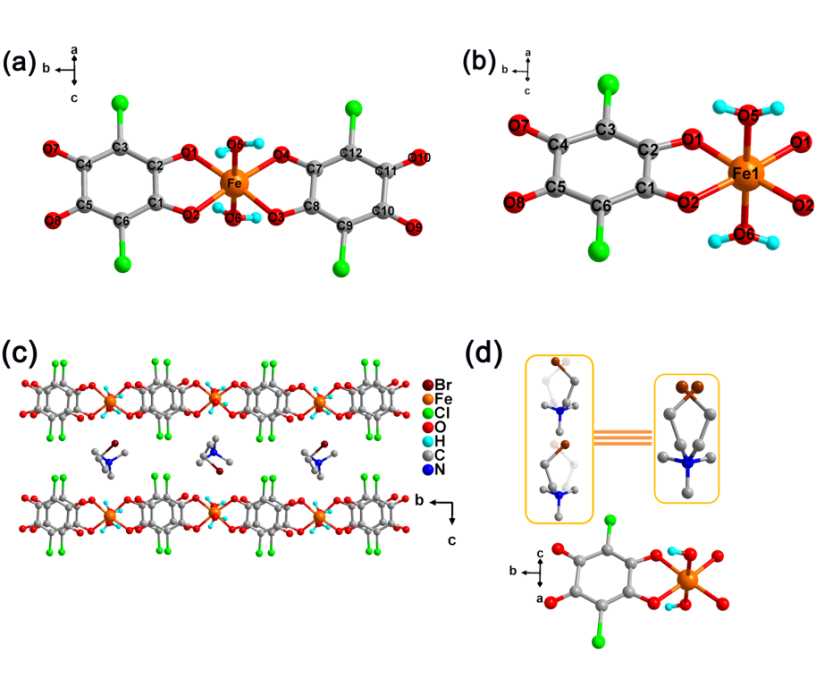


Figure S2. The atomic label of **1** at 100 K (a) and 300 K (b). Assembled structure of **1** along the *a*-axis (c). The asymmetric unit of **1** at 300 K (d).





Figure S3. Infrared spectra of 1 at room temperature.

**

**

Figure S4. EPR spectra of 1 recorded from 100 K to 390 K.


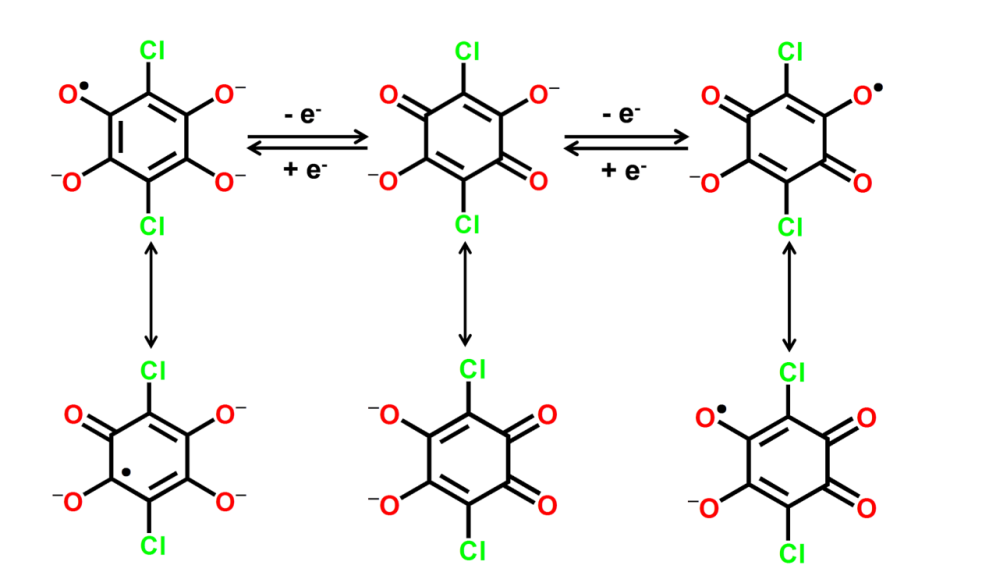


**Figure S5.** Redox series of chloranilic acid: left to right, Cl_2_An^3−•^, Cl_2_An^2−^, Cl_2_An^1−•^.





**Figure S6**. UV-Vis-NIR spectra of **1** at room temperature.

**Table S3.** The Mössbauer fitting parameters for **1**.

| Fe^III^-*hs* | | | | | | Fe^II^-*hs* | | | |
| --- | --- | --- | --- | --- | --- | --- | --- | --- | --- |
|  | T / K | IS  / mm s^-1^ | QS  / mm s^-1^ | LW  / mm s^-1^ | Ratio  (%) | IS  / mm s^-1^ | QS  / mm s^-1^ | LW  / mm s^-1^ | Ratio  (%) |
| electron  transfer  relaxation  model | 100 | 0.48 | 1.32 | 0.36 | 96.7 | 1.08^*^ | 2.11^*^ | 0.36 | 3.3 |
|  | 200 | 0.43 | 1.30 | 0.41 | 96.0 | 1.08^*^ | 2.11^*^ | 0.41 | 4.0 |
|  | 250 | 0.39 | 1.33 | 0.38 | 93.9 | 1.08^*^ | 2.11^*^ | 0.38 | 6.1 |
|  | 300 | 0.38 | 1.39 | 0.34 | 91.2 | 1.08^*^ | 2.11^*^ | 0.34 | 8.8 |
|  | 370 | 0.24 | 1.32^*^ | 0.38 | 86.9 | 1.08^*^ | 2.11^*^ | 0.38 | 13.1 |

^*^These parameters were fixed at the value obtained for the spectrum at the nearest temperature.


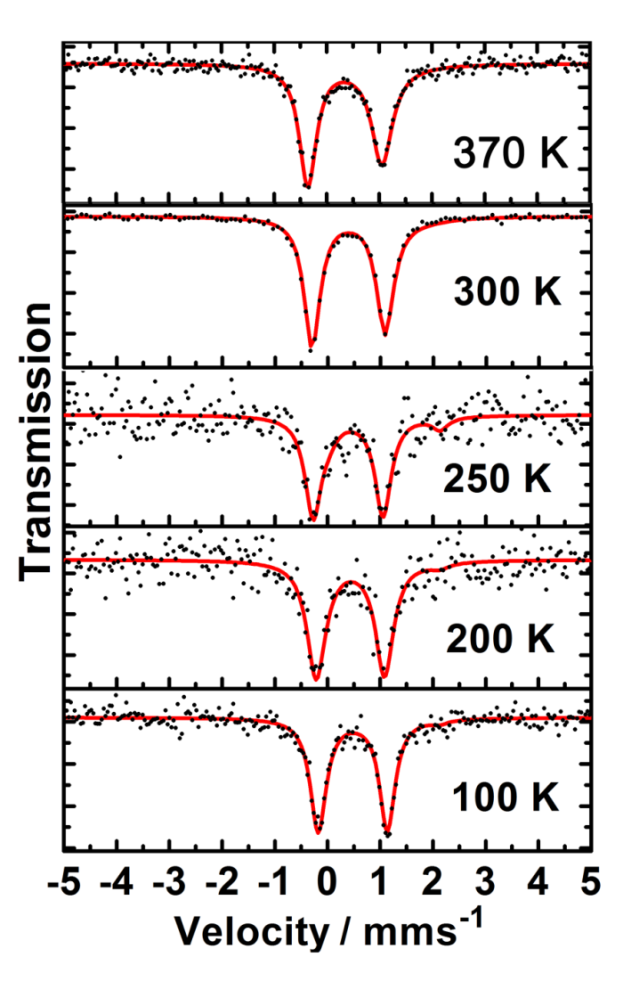


**Figure S7.** The Mössbauer spectra for **1** from 100 K to 370 K, with the fitting spectrum shown in red.


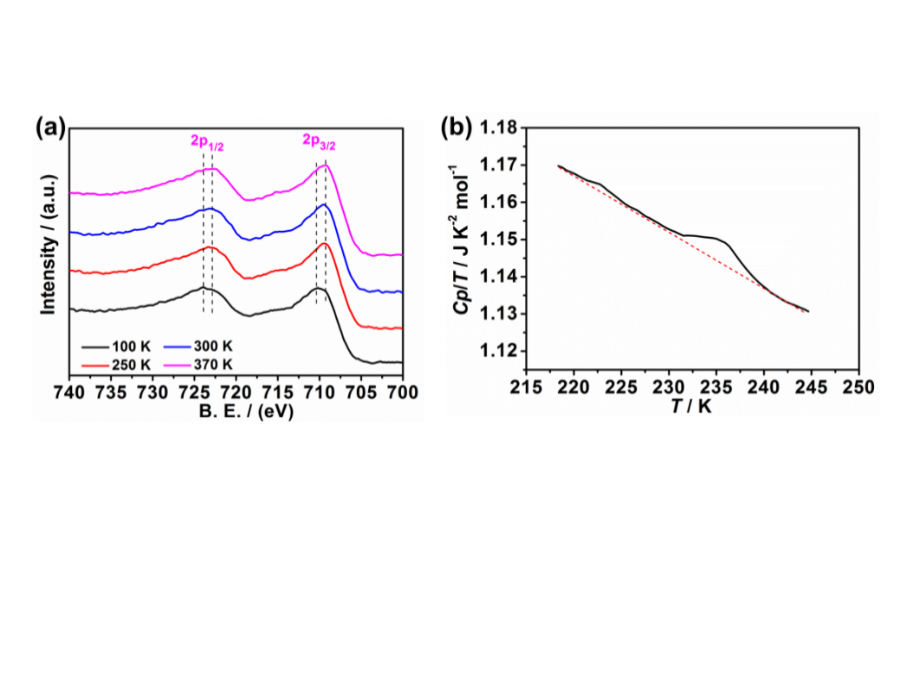


Figure S8. (a) Temperature-dependent XPS survey spectrum of 1 from 100 to 370 K. (b) The heat capacity of **1**.


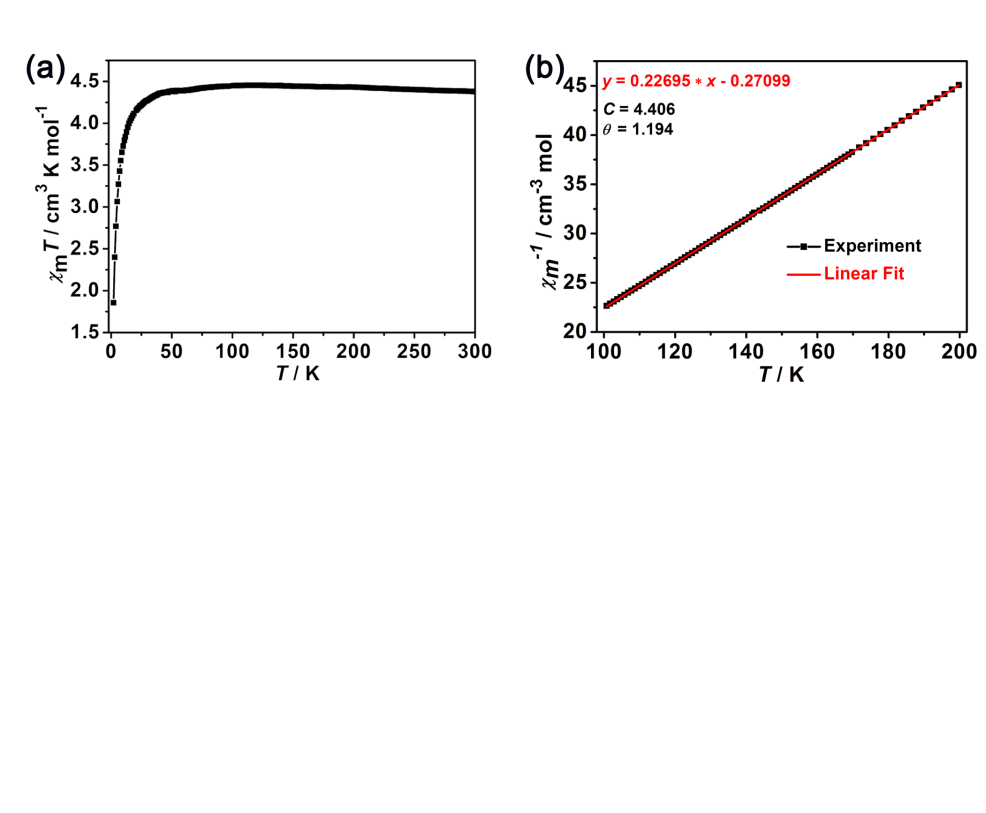


Figure S9. (a) Temperature-dependent magnetic susceptibility of **1**. (b) Plots of 1/*χ*_m_ vs. temperature and the linear fitting according to the Curie-Weiss Law.

**
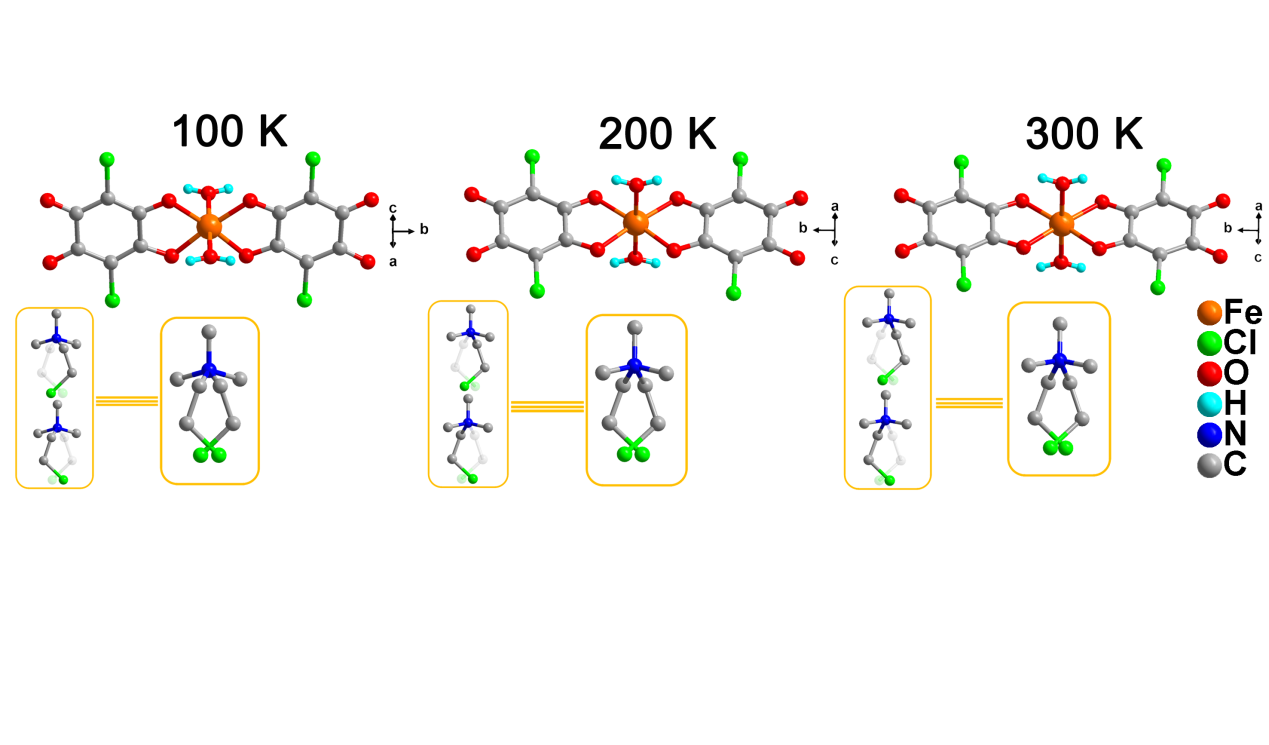
**

**Figure S10.** Crystal structure of **2** at 100 K, 200 K and 300 K.





**Figure S11.** EPR spectra of **2** in the temperature range of 100‒390 K.

**

**

**Figure S12.** Temperature-dependent dielectric constant of **2** at different frequencies.


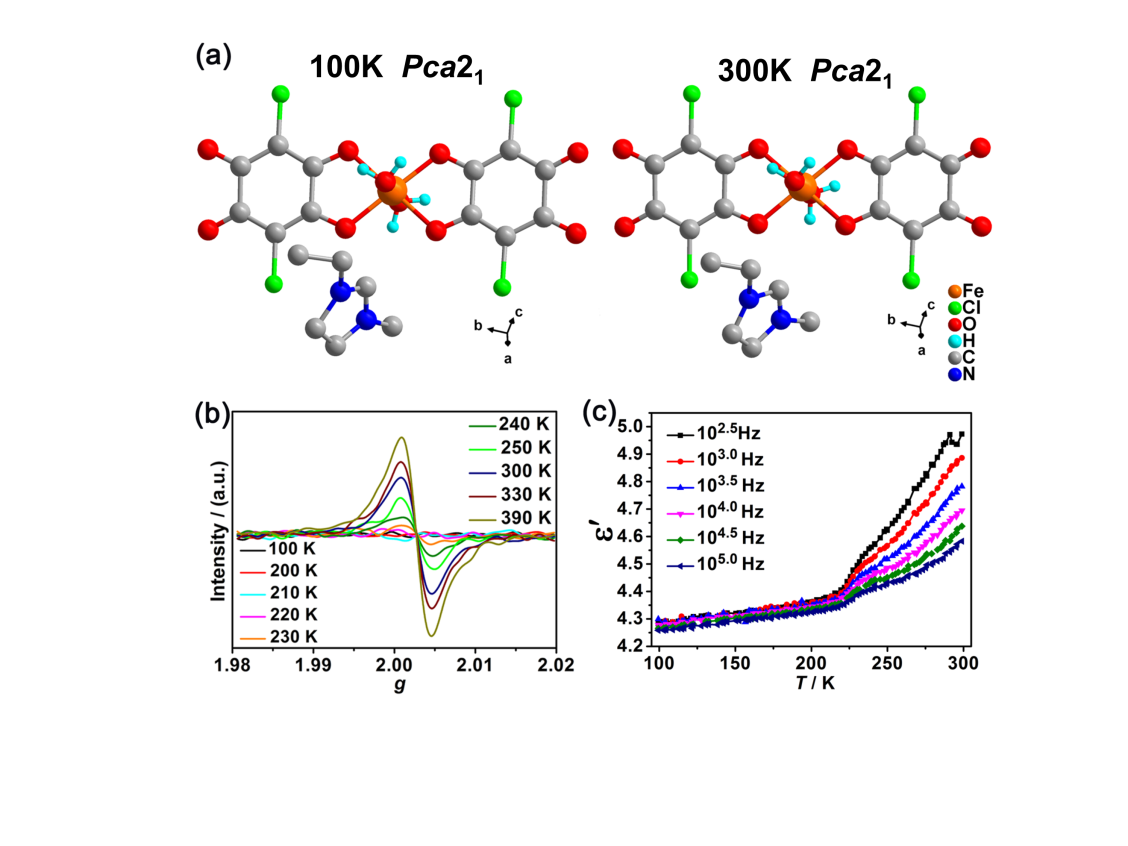


**Figure S13.** (a) Crystal structure of **3** at 100 K and 300 K. (b) The temperature-dependence of EPR of **3**. (c) Temperature-dependence of dielectric constant at different frequencies of **3**.


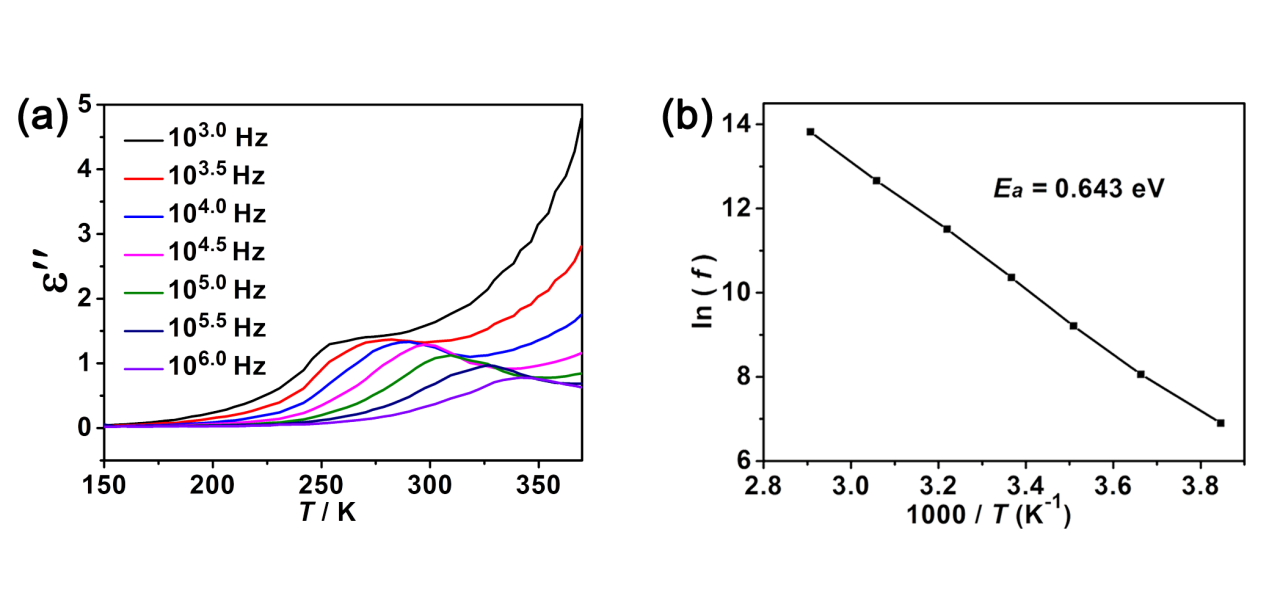


**Figure S14.** Values of the imaginary part (*ε″*) of the dielectric constant at various frequencies and temperatures (a) and (b) linear fitting of ln(*f*) versus 1000/*T* for **1**.


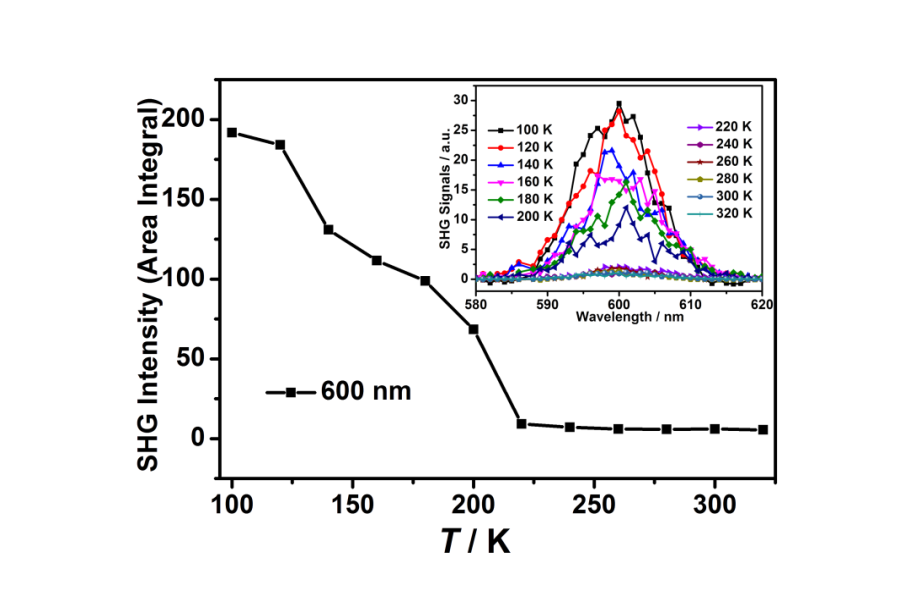


**Figure S15.** The temperature-dependence of SHG for **1**.


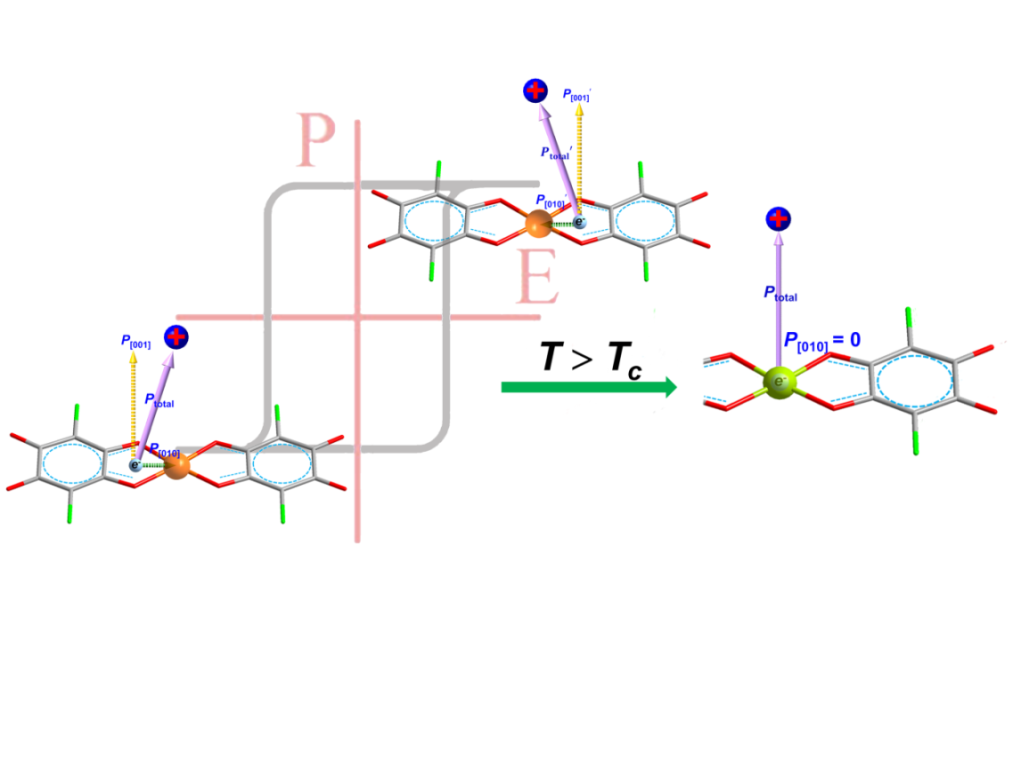


**Figure S16.** Schematic representation of the intrinsic spontaneous polarization of the ferroelectric phase of **1**.

The detailed calculation of ferroelectric polarization is as follows. The polarization (*P*) can be estimated using the following method:

*P* = *μ* / *V*; *μ* = *q* • *d*;

where *μ* is dipole moment, *d* is the effective distance of the electron transfer, *q* is the number of transferring electrons, *V* is the volume of **1**. ^57^Fe Mössbauer spectra indicate that **1** contains 87% of Fe^III^ and 13% Fe^II^, and the percentage of charge transfer is 13%. Combing with crystal data and Mössbauer spectra, *d* = 1.77 × 10^-11^m; Cell parameters: *a* = 7.06460 Å, *b* = 17.0210 Å, *c* = 9.5030 Å, V = 1116.05 Å^3^, Z = 2.

Therefore, *μ* = 2e•*d* = 2 × 1.6 × 10^-19^ × 13% ×1.77 × 10^-11^ C•m ≈ 7.36 × 10^-31^ C•m; and *P* = 7.36 × 10^-31^ C•m / 1116.05 × 10^-30^ m^3^ ≈ 65.9 nC/cm^2^.





**Figure S17**. Temperature-dependent electric polarization of **3**.


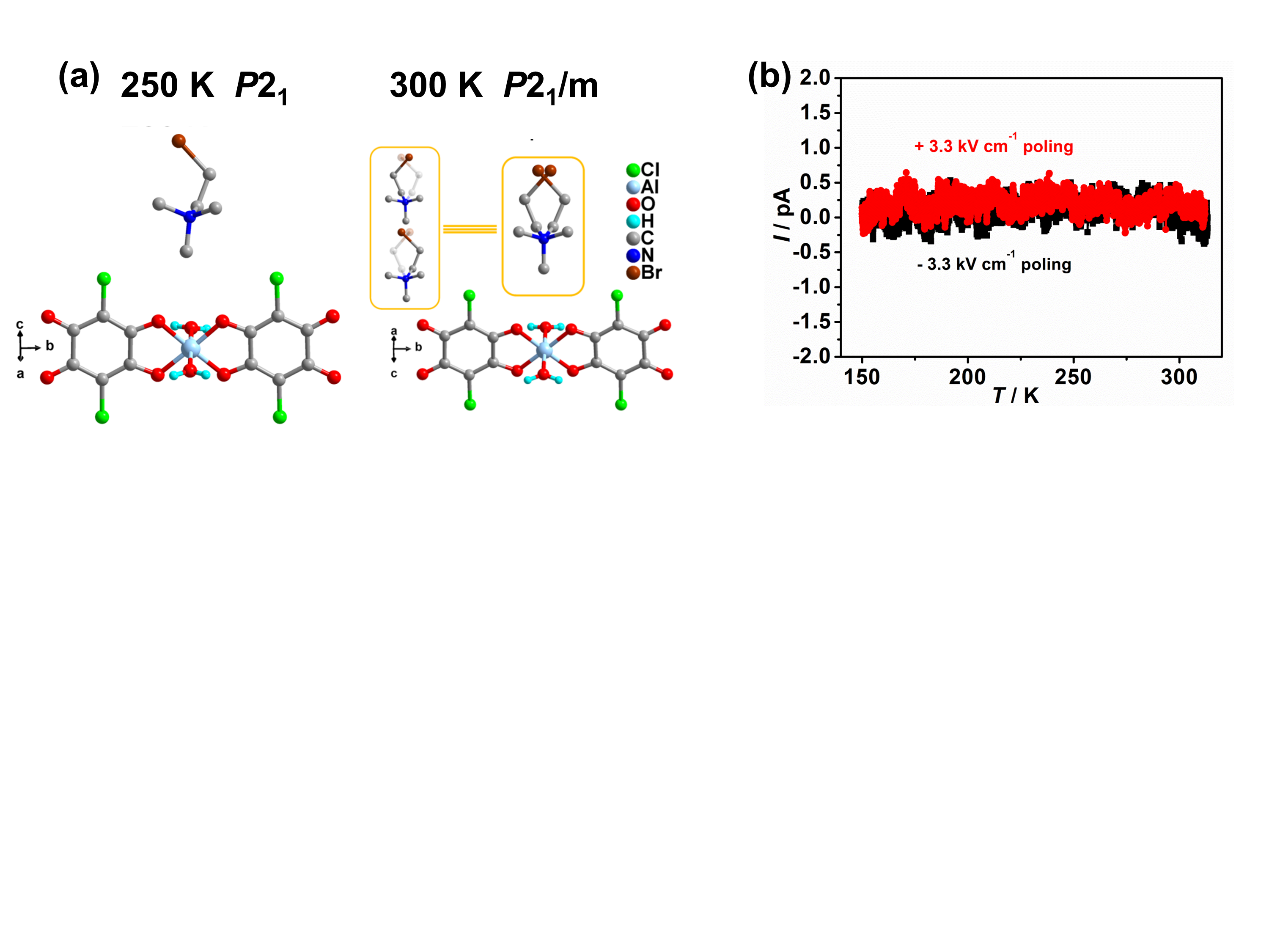


**Figure S18.** (a) Crystal structure of **4** at 250 K (order phase) and 300 K (disorder phase). (b) The temperature dependence of pyroelectric current of **4**.


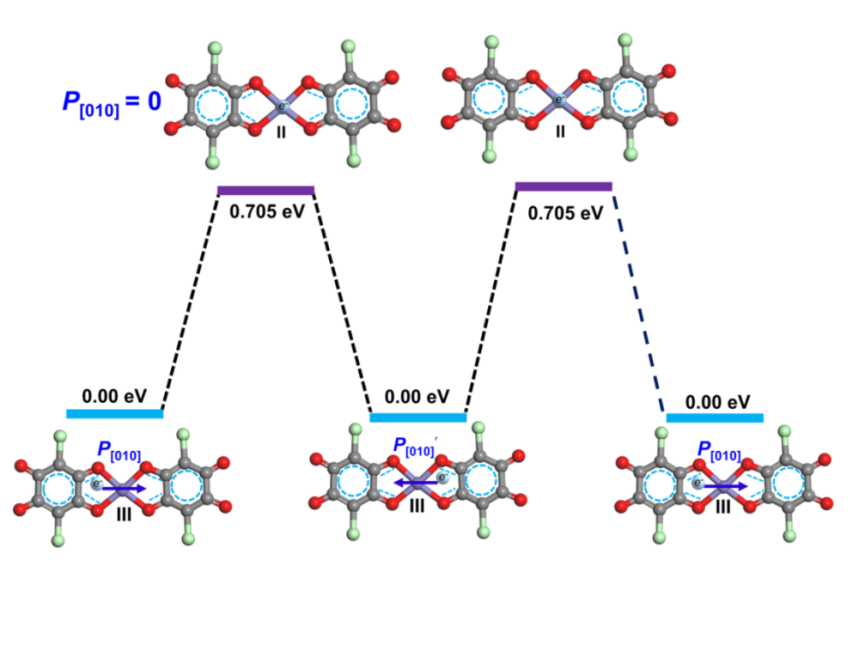


**Figure S19.** Scheme for path of polarization reversal of **1**.

**
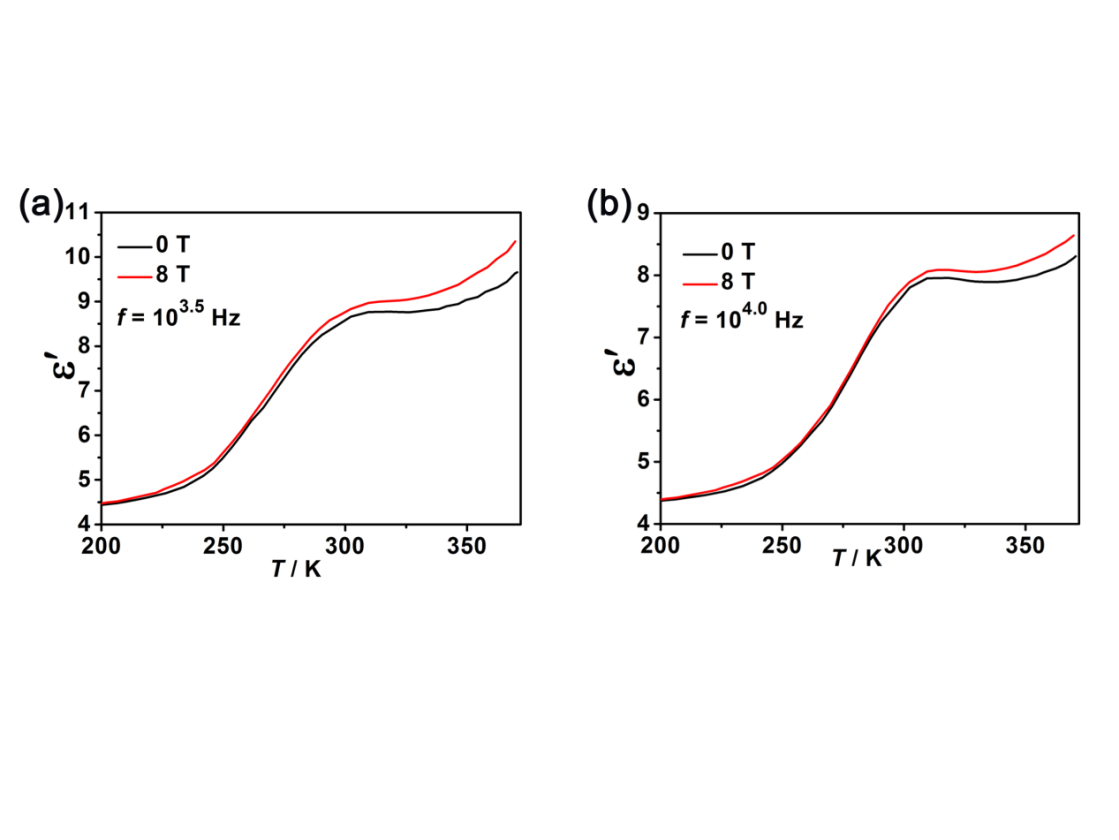
**

**Figure S20.** Dielectric constant versus temperature at 10^3.5^ Hz (a) and 10^4.0^ Hz (b), measured at zero field and under an external magnetic field of 8 T of **1**.





**Figure 21.** The electric polarization of **3** at different magnetic fields.





**Figure 22.** Dielectric loss versus temperature of **1** at 10^3.0^ Hz measured in zero field and in an external magnetic field of 8 T.


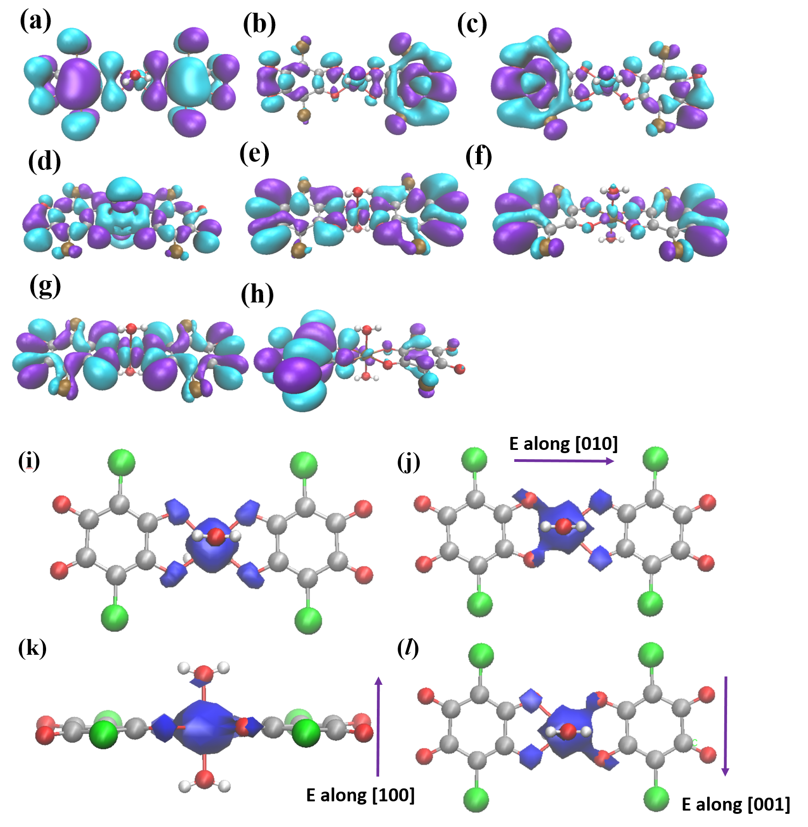


**Figure S23.** (a-c) Double occupied molecular orbitals (DOMO), (d-h) single-occupied molecular orbitals (SOMO), spin density at zero field (i) and electric field of 0.07 V/nm along [010] (j), [100] (k) and [001] (l) of **1**. **Electronic-structure description**: From the frontier orbital characteristic with *C*_2h_ symmetry, **1** features electronic configuration of (a_u_)^2^(b_u_)^2^(b_g_)^2^(a_g_)^1^(b_g_)^1^(a_u_)^1^(a_g_)^1^(a_u_)^1^. Three DOMOs (a-c) mainly involve in the anti-bonding π-type orbital of ligand and tiny 3*d* orbitals of Fe ion. For five SOMOs, three SOMOs ((d), (e) and (g)) with irreducible representation of A_g_, B_g_ and A_g_ mainly concentrates on anti-bonding π-type orbital of ligand and 3*d* orbitals (d_z2_, d_xy_, d_x2-y2_ of Fe ion), while two SOMOs ((f) and (h)) with the same irreducible representation of A_u_ mainly consist of anti-bonding π-type orbital of ligands. Owing to the inversion-symmetry characteristic of 3*d* orbitals, two SOMO with A_u_ representation is impossible to contain 3*d* orbitals of Fe ion. Thus, it is supposed that the electronic transfers in *C*_2h_ symmetry may occurs from DOMO a_u_ or b_u_ orbitals to the a_g_ SOMO under the transition operator of A_u_ and B_u_: a_u_ → a_g_ and b_u_ → a_g_.


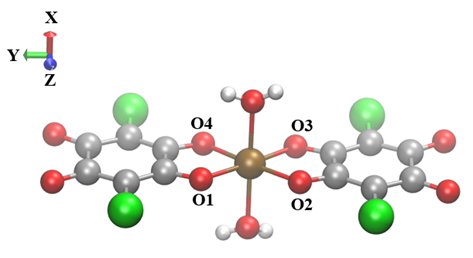


**Figure S24.** Atomic label and coordination system in non-collinear calculation.

**Table S4.** Euler angle (theta, phi) of magnetic moment from non-collinear DFT calculation (unit: deg.). The atomic label and coordination system were presented in Figure S24.

| Atom | Spin moment | Orbit moment |
| --- | --- | --- |
| Fe | (2.61, 177.66) | (7.33, 176.00) |
| O1 | (12.86, 101.60) | (44.88, 267.37) |
| O2 | (12.34, 256.17) | (50.15, 91.15) |
| O3 | (9.65, 105.62) | (52.65, 267.61) |
| O4 | (9.59, 251.58) | (56.40, 92.88) |


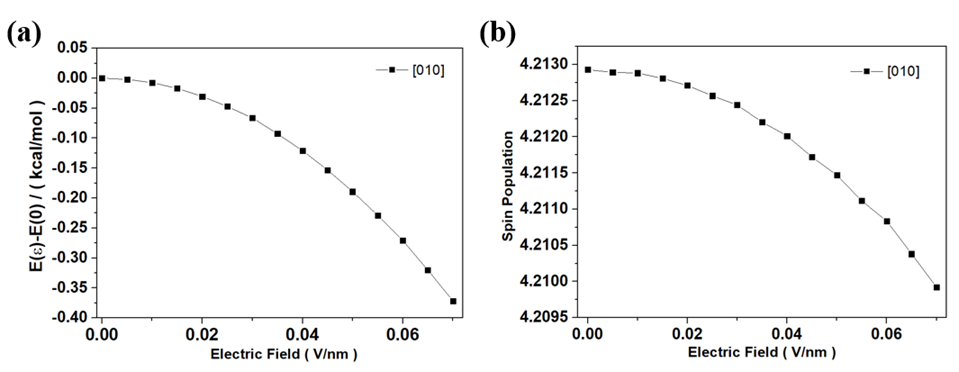


**Figure S25.** Electric-field dependence properties of Fe(II)-based **1** along [010] direction: Energy difference (a) and the population of spin magnetic moment for Fe ion (b).


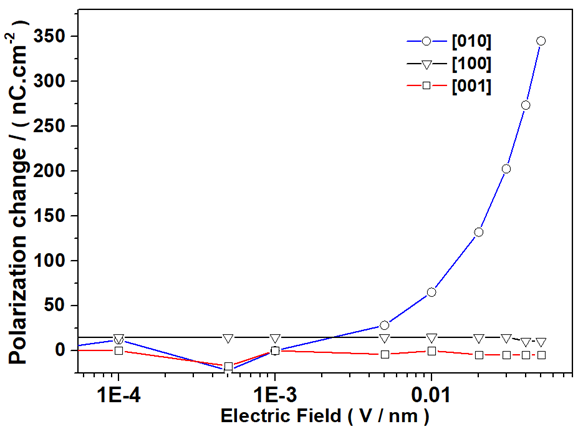


**Figure S26**. Applied electric field induced electronic polarization change of Fe(II)-based **1** along [010], [100] and [001] lattice direction.


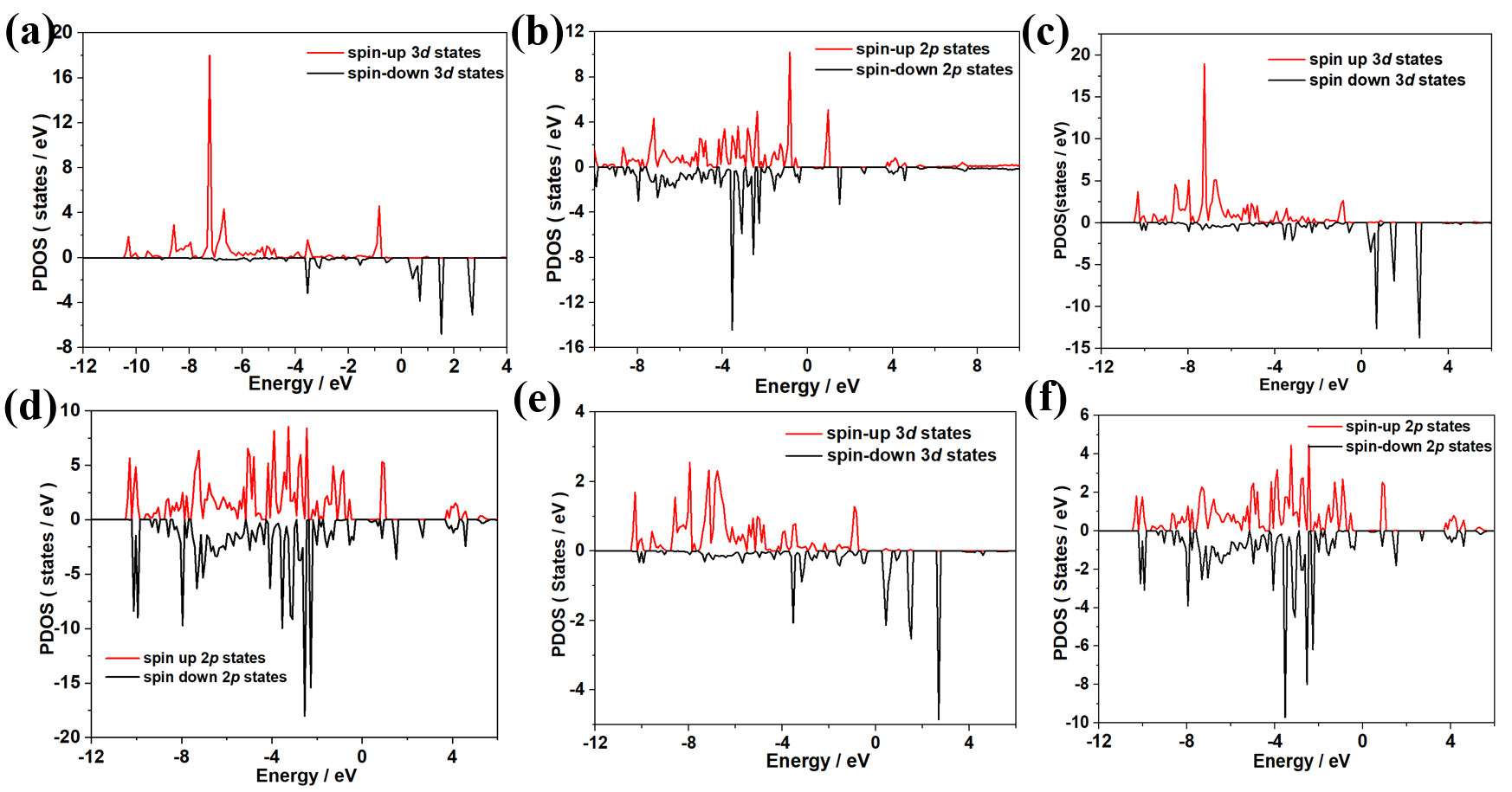


**Figure S27.** Partial Density of states under the zero field (a-b), magnetic field of 8 T (c-d) and electric field of 0.07 V/nm (e-f) with the 3*d* states of Fe and 2*p* states of coordinated O.


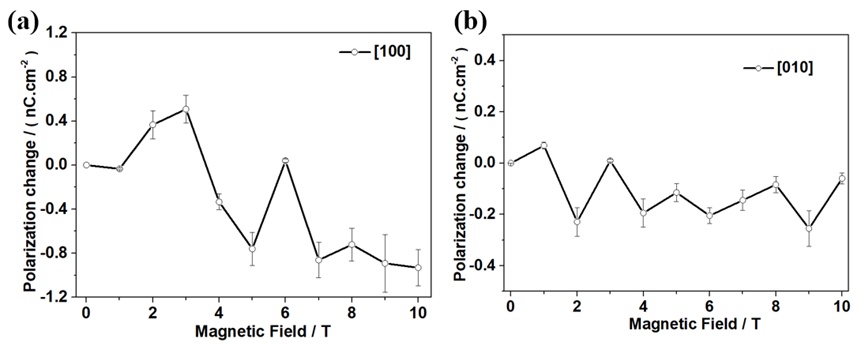


**Figure S28**. Applied magnetic field induced electronic polarization change of Fe(II)-based **1** along [100] (a) and [010] (b) lattice direction.


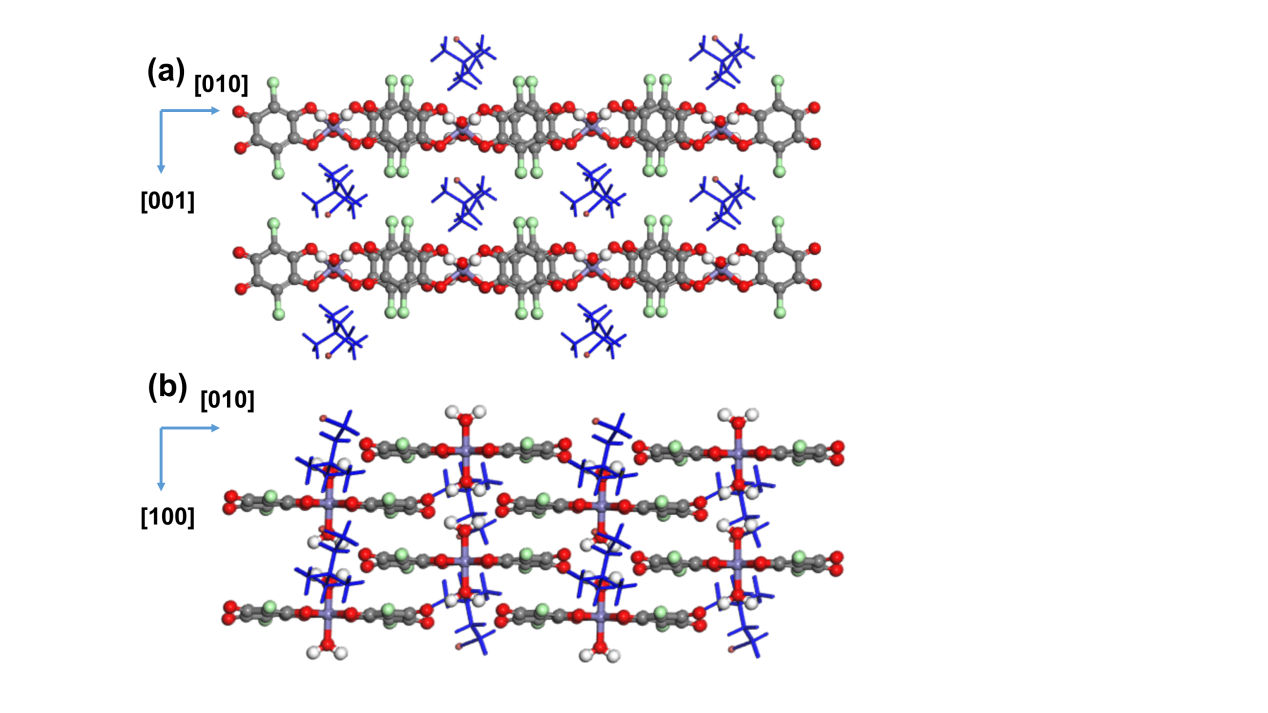


**Figure S29.** Two views of relaxed structure along different lattice direction.


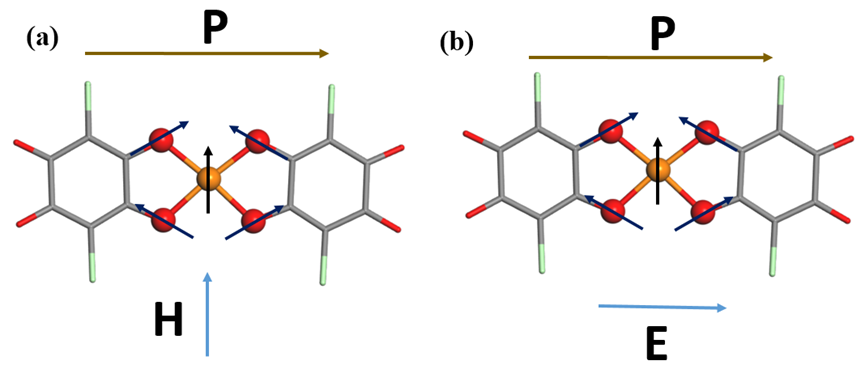


**Figure S30.** Schematic represention of magnetoelectric effect mechanism of **1**.

**Computational details**

**Calculation of Magnetic-Field Effect**: Spin-polarized density functional theory calculations were performed using the ABINIT program.^1^ The exchange-correlation effect in Kohn-shame equation was described by Perdew-Burke-Ernzerhof functional with the generalized gradient approximation (GGA) following inhomogeneous charge-density distributions.^2^ In the treatment of inner core and valent electrons, Projector-Augmented Wave (PAW) method was used to treated. PAW features the accuracy of all-electron method as well as the efficiency of pseudopotential method.^3^ The cutoff energy of 500 eV is set to expansion of plane wave function basic set. The 8× 4× 8 grid with Г-pointer center was used to sample during Brillouin zone, based on Monkhorst-Pack method.^4^ To account for the strong Coulomb repulsion of metal ions and reduce the self-interaction error from PBE functional, DFT+U strategy was used to correct the on-site Coulomb repulsions with the *U* value of 4.0 eV and *J* of 1.0 eV,^5^ which accurately describes the electronic transfer of Fe ions.^6^ The tolerance for self-consistent field energy calculation and geometrical optimization were set to 1×10^-6^ eV and 5.0×10^-4^ Ha/Bohr, respectively. To achieve accurate weak interaction between adsorption species and MOF, the Van der Waals correction method (DFT-vdW) was adopted for all self-consistent field calculations.^7^ The initial structures are derived from X-ray single crystal diffraction at 100 K and 200 K, where the atomic positions of hydrogen atoms were fully relaxed without any restrictions. According to modern theory of polarization introduced by King-Smith and Vanderbilt and computational method developed by E. Bousquet and K. T. Delaney,^8,9^ applied magnetic field induced polarization response were investigated by using a self-consistent response to a Zeeman field for noncollinear spins.

**Calculation of Electric-Field Effect and Non-Collinear Property:** Spin-polarized DFT calculations with the exchange-correlation functional of PBE in the framework of generalized gradient approximation, where non-collinear and spin-orbit coupling computation were taken into account, were performed by using OpenMX 3.9 program.^10-12^ Electronic wave functions are expanded by using a linear combination of multiple pseudoatomic orbitals (LCPAOs), and inner core electrons were treated by norm-conserving pseudopotentials. A mesh of 8× 4× 8 grid under the Monkhorst-Pack scheme,^4^ which had been tested by convergence test, was sampled in the integration over the Brillouin zone. Residual minimization method in the direct inversion iterative subspace (RMM-DIIS) was used to enhance the SCF convergence with the energy cutoff of 400 Ry and convergence criterion of 1×10^-8^ Ha.^13^ Based on DFT-U computational strategy,^5^ the U value of 4.0 eV and J of 1.0 eV were used described on-site Coulomb repulsions.

**Multi-reference Calculation and Broken-Symmetry Calculation:** The scalar relativistic Douglas–Kroll–Hess (DKH) Hamiltonian was used with DKH-def2-TZVP basic set for all atoms.^14^ A combination of the states-averaged complete active space self-consistent field (CASSCF) and second order N-electron valence state pertubation theory (NEVPT2) for dynamic correlation,^15,16^ together with quasi-degenerate perturbation theory (QDPT) method,^17^ were used to calculate magnetic parameters. Spin-orbital coupling was taken into account in the anisotropy relativistic calculation. RI approximation was used to deal with fock operator in CASSCF and NEVPT2 calculations. In SCF calculations, the keywords of Grid5 and very-tight convergence setting were used to improve the computational accuracy. For Fe(III) ion, we considered all of roots in the CAS(5, 5) active space with 5 electrons in 5 3*d*-metal orbitals, consisting of 1 root for *S* = 5/2, 24 roots for *S* = 3/2 and 75 roots for *S* = 1/2. For the configuration of Fe(II) and radical ions, we referred to the roots of CAS(7,6) active space with 7 electrons in 5 3*d*-metal orbitals and 1 π-type 2*p* orbital, consisting of 1 root of all 6 roots for *S* = 5/2, 48 roots of all 84 roots for *S* = 3/2 and all 210 roots for *S* = 1/2. Moreover, magnetic coupling parameters was evaluated by using symmetry-breaking calculations.^18^ Computational model of cluster were derived from aforementioned computational result at 100K and 200K without any geometrical optimization. Generally, these calculations were performed with electronic structure program package of ORCA 4.2.1.^19^

**CDFT calculations**: All constraint density function theory (CDFT) calculations were conducted using the spin dependent formalism of the hybrid Gaussian and (augmented) plane waves method.^20^ Exchange−correlation term was described by PBE functional. Valence electrons were treated by molecularly optimized Gaussian basis sets of double ζ plus polarization quality (MOLOPT-DZVP-SR),^21^ and inner cores were treated by norm conserving GTH-pseudopotentials.^22^ DFT-D3 van der Waals corrections was used to correct weak interaction.^23^ In all calculations of SCF, the orbital transformation minimizer was used to promote the convergence.^24^ During CDFT calculations, the constraints were defined by Becke weight adjusted with covalent atomic radii (BW+A).^25^ The constraints were optimized using the Newton-Raphson method with backtracking line search and an initial step size *α* = 1. And the Jacobian matrix is calculated on each iteration by sequentially perturbing each constraint Lagrangian ξj by 5×10^−3^ and minimizing SCF energy. Based on DFT-U computational strategy,^5^ the U value of 4.0 eV and J of 1.0 eV were used described on-site Coulomb repulsions of Fe ions. These calculations were carried out by using CP2K 7.1 program.^26,27^

**Reference**

1. Gonze X, Amadon B and Anglade PM *et al.* ABINIT: First-principles approach to material and nanosystem properties. *Comp Phys Commun* 2009; **180**: 2582-2615.

2. Perdew J, Burke K and Ernzerhof M. Generalized gradient approximation made simple. *Phys Rev Lett* 1996; **77**: 3865-3868.

3. Kresse G, Furthmüller J. Efficiency of *ab*-initio total energy calculations for metals and semiconductors using a plane-wave basis set. *Comput Mater Sci* 1996; **6**: 15-50.

4. Monkhorst HJ, Pack JD. Special points for Brillonin-zone integrations. *Phys Rev B* 1976; **13**: 5188-5192.

5. Himmetoglu B, Floris A and De Gironcoli, S *et al.* M. Hubbard-corrected DFT energy functionals: The LDA+U description of correlated systems. *Int J Quantum Chem* 2014; **114**: 14-49.

6. Collman JP, Hoard JL and Kim N *et al.* Synthesis, Stereochemistry, and Structure-Related Properties of α, β, γ, δ-Tetraphenylporphinatoiron(II). *J Am Chem Soc* 1975; **97**: 2676-2681.

7. Grimme S, Antony J and Ehrlich S *et al.* A consistent and accurate *ab* initio parametrization of density functional dispersion correction (DFT-D) for the 94 elements H-Pu. *J Chem Phys* 2010; **132**:154104.

8. King-Smith R. D, Vanderbilt D. Theory of polarization of crystalline solids. *Phys Rev B* 1993; **47**: 1651.

9. Bousquet E, Spaldin NA and Delaney KT. Unexpectedly Large Electronic Contribution to Linear Magnetoelectricity. *Phys Rev Lett* 2011; **106**: 107202.

10. Ozaki T. Variationally optimized atomic orbitals for large-scale electronic structures. *Phys Rev B* 2003; **67**: 155108.

11. Ozaki T, Kino H. Numerical atomic basis orbitals from H to Kr. *Phys Rev B* 2004; **69**: 195113.

12. Ozaki T, Kino H. Efficient projector expansion for the *ab* initio LCAO method. *Phys Rev B* 2005; **72**: 045121.

13. Pulay P. Convergence acceleration of iterative sequences. the case of scf iteration. *Chem Phys Lett* 1980; **73**: 393–398*.*

14. Pantazis DA, Neese F. All-Electron Scalar Relativistic Basis Sets for the Actinides. *J Chem Theory Comput* 2011;**7**: 677–684.

15. Hegarty D, Robb MA. Application of unitary group methods to configuration interaction calculations. *Mol Phys* 1979; **38**: 1795-812.

16. Angeli C, Cimiraglia R and Evangelisti S *et al.* Introduction of *n*-electron valence states for multireference perturbation theory. *J Chem Phys* 2001; **114**: 10252.

17. Souza B, Farias G and Neese F *et al.* Predicting Phosphorescence Rates of Light Organic Molecules Using Time-Dependent Density Functional Theory and the Path Integral Approach to Dynamics. *J Chem Theory Comput* 2019; **15**: 1896.

18. Bencini A, Totti F. A Few Comments on the Application of Density Functional Theory to the Calculation of the Magnetic Structure of Oligo-Nuclear Transition Metal Clusters. *J Chem Theory Comput* 2009; **5**: 144.

19. Neese F. Software update: the ORCA program system, version 4.0*. Comput Mol Sci WIREs* 2018; **8**: e1327.

20. Lippert G, Hutter J and Parrinello M. A hybrid Gaussian and plane wave density functional scheme. *Mol Phys* 1997; **92**: 477−487.

21. Vondele VJ, Hutter J. Gaussian basis sets for accurate calculations on molecular systems in gas and condensed phases. *J Chem Phys* 2017; **127**: 114105.

22. Goedecker S, Teter M and Hutter J. Separable dual-space Gaussian pseudopotentials. *Phys Rev B* 1996; **54**: 1703−1710.

23. Grimme S, Antony J and Ehrlich S *et al.* A consistent and accurate ab initio parametrization of density functional dispersion correction (DFT-D) for the 94 elements H-Pu. *J Chem Phys* 2010; **132**: 154104.

24. Vondele VJ, Hutter J. An efficient orbital transformation method for electronic structure calculations. *J Chem Phys* 2003; **118**: 4365−4369.

25. Becke AD. A multicenter numerical integration scheme for polyatomic molecules. *J Chem Phys* 1988; **88**: 2547−2553.

26. Vondele VJ, Krack M and Mohamed F *et al.* Quickstep: Fast and accurate density functional calculations using a mixed Gaussian and plane waves approach. *Comput Phys Commun* 2005; **167**: 103−128.

27. Hutter J, Iannuzzi M and Schiffmann F *et al.* CP2K: atomistic simulations of condensed matter systems. *WIREs Comput Mol Sci* 2014; **4**: 15−25.
